# A Microsaccadic Account of Attentional Capture and Inhibition of Return in Posner Cueing

**DOI:** 10.3389/fnsys.2016.00023

**Published:** 2016-03-07

**Authors:** Xiaoguang Tian, Masatoshi Yoshida, Ziad M. Hafed

**Affiliations:** ^1^Physiology of Active Vision Laboratory, Werner Reichardt Centre for Integrative Neuroscience, University of TuebingenTuebingen, Germany; ^2^Graduate School of Neural and Behavioural Sciences, International Max-Planck Research School, University of TuebingenTuebingen, Germany; ^3^Department of Developmental Physiology, National Institute for Physiological SciencesOkazaki, Japan

**Keywords:** microsaccades, fixational eye movements, covert visual attention, Posner cueing, retinal-image stabilization, attentional capture, inhibition of return

## Abstract

Microsaccades exhibit systematic oscillations in direction after spatial cueing, and these oscillations correlate with facilitatory and inhibitory changes in behavioral performance in the same tasks. However, independent of cueing, facilitatory and inhibitory changes in visual sensitivity also arise pre-microsaccadically. Given such pre-microsaccadic modulation, an imperative question to ask becomes: how much of task performance in spatial cueing may be attributable to these peri-movement changes in visual sensitivity? To investigate this question, we adopted a theoretical approach. We developed a minimalist model in which: (1) microsaccades are repetitively generated using a rise-to-threshold mechanism, and (2) pre-microsaccadic target onset is associated with direction-dependent modulation of visual sensitivity, as found experimentally. We asked whether such a model alone is sufficient to account for performance dynamics in spatial cueing. Our model not only explained fine-scale microsaccade frequency and direction modulations after spatial cueing, but it also generated classic facilitatory (i.e., attentional capture) and inhibitory [i.e., inhibition of return (IOR)] effects of the cue on behavioral performance. According to the model, cues reflexively reset the oculomotor system, which unmasks oscillatory processes underlying microsaccade generation; once these oscillatory processes are unmasked, “attentional capture” and “IOR” become direct outcomes of pre-microsaccadic enhancement or suppression, respectively. Interestingly, our model predicted that facilitatory and inhibitory effects on behavior should appear as a function of target onset relative to microsaccades even without prior cues. We experimentally validated this prediction for both saccadic and manual responses. We also established a potential causal mechanism for the microsaccadic oscillatory processes hypothesized by our model. We used retinal-image stabilization to experimentally control instantaneous foveal motor error during the presentation of peripheral cues, and we found that post-cue microsaccadic oscillations were severely disrupted. This suggests that microsaccades in spatial cueing tasks reflect active oculomotor correction of foveal motor error, rather than presumed oscillatory covert attentional processes. Taken together, our results demonstrate that peri-microsaccadic changes in vision can go a long way in accounting for some classic behavioral phenomena.

## Introduction

Microsaccades are small saccades that occur repeatedly during prolonged fixation (Hafed, 2011; Hafed et al., 2015). Recent results have demonstrated that microsaccade generation is associated with substantial changes in visual sensitivity and perceptual performance, and that these changes are directionally dependent in the pre-movement interval (Hafed, 2013; Chen et al., 2015; Hafed et al., 2015). Even though the mechanisms behind these changes are not yet fully elucidated (Hafed et al., 2015), an important question arises out of them nonetheless. Specifically, because microsaccades inescapably occur in a variety of experiments enforcing fixation, one wonders how large of a contribution these microsaccade-related changes in vision have in such experiments? It could be the case that these movements are rare enough to be completely inconsequential, or it could be the case that active peri-microsaccadic changes in vision play a substantial role, despite the diminutive size of the eye movements, much like active peri-saccadic changes in vision (for large saccades) can dramatically alter the state of the visual system (Duhamel et al., 1992; Cai et al., 1997; Ross et al., 1997, 2001; Lappe et al., 2000; Tolias et al., 2001; Sommer and Wurtz, 2006; Pola, 2011; Morris et al., 2012, 2013; Zirnsak et al., 2014).

In this study, we used a theoretical approach, motivated by a recent hypothesis (Hafed et al., 2015), in order to investigate the above question. We chose as a case study the Posner cueing task, which had been used to great effect in advancing our understanding of covert visual attention (Posner, 1980). In this task, spatial cueing facilitates stimulus detection at the cued location relative to other locations (Posner, 1980). However, cueing eventually leads to slower reaction times (RT’s; Posner, 1980; Posner and Cohen, 1984; Posner et al., 1985). This latter phenomenon, termed “inhibition of return” (IOR; Posner and Cohen, 1984; Posner et al., 1985; Klein, 2000; Lupianez et al., 2006), was thought to reflect a cognitive mechanism through which “the nervous system favors novel information over information previously presented” (Posner et al., 1985).

We elected to investigate Posner cueing because microsaccades are robustly modulated in it (Hafed and Clark, 2002; Engbert and Kliegl, 2003; Hafed et al., 2011, 2013; Hafed and Ignashchenkova, 2013). In fact, cue onset causes modulations in microsaccades that are so robust that they can occur even after tens of thousands of trials performed by an individual subject (Hafed et al., 2011, 2015; Hafed and Ignashchenkova, 2013). This suggests that these modulations are highly reflexive, much like how a brief, irrelevant flash reflexively alters saccade onset time distributions (Reingold and Stampe, 2002). Consistent with this, a model in which microsaccades repeatedly occur, and in which these eye movements are reflexively reset by stimulus transients, is sufficient to replicate observations of how microsaccades are modulated after cue onset (Hafed and Ignashchenkova, 2013).

**FIGURE 1:**
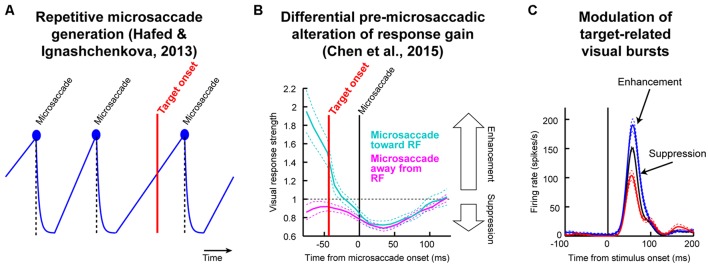
**Testing the theoretical implications of pre-microsaccadic changes in visual sensitivity. (A)** During prolonged gaze fixation, microsaccades repetitively occur. This can be modeled as a repetitive rise-to-threshold process (Hafed and Ignashchenkova, 2013). If a peripheral target were to appear at a random time, it would necessarily appear at different times relative to microsaccade onset. **(B)** Peripheral target onset near microsaccades experiences altered visual sensitivity (Chen et al., 2015). Importantly, this effect is directionally dependent in the pre-movement interval (Hafed, 2013; Chen et al., 2015), such that visual sensitivity is enhanced if a target appears congruent with the upcoming microsaccade direction and suppressed if it is opposite the upcoming microsaccade. **(C)** The net result is that for an identical target onset, visual bursts can be either enhanced or suppressed as a function of microsaccade direction. Since response gain strength is a direct determinant of behavioral performance (Hafed et al., 2015), particularly for both saccadic and manual reaction times (RT’s), an important theoretical question to ask is: are these microsaccade-related changes sufficient to account for classic behavioral phenomena? In this study, we used a theoretical approach to investigate this issue for a specific type of task. We developed a model only implementing the two concepts in this figure (microsaccade repetitiveness and differential pre-microsaccadic changes in vision) and asked how much explanatory power it has. **(B,C)** Were modified with permission from (Chen et al., 2015).

As we elucidate below, resetting of the oculomotor system by cue onset means that any systematic processes underlying microsaccade generation become unmasked by the cue (Hafed and Ignashchenkova, 2013; Hafed et al., 2015). Now, imagine that after such unmasking, a second stimulus comes (e.g., the post-cue target in Posner cueing). This second stimulus will necessarily arrive at the visual system at a specific time relative to microsaccade onset (**Figure 1A**). Because of pre-microsaccadic alteration in visual sensitivity (**Figure 1B**), target-related visual bursts (**Figure 1C**) will therefore be either enhanced or suppressed (for the exact same target) depending on whether they arrive congruent or incongruent with upcoming microsaccade direction. Because microsaccades have systematic post-cue directions (Hafed and Ignashchenkova, 2013), then a theoretically plausible possibility is that repetitive microsaccades (**Figure 1A**), coupled with known differential microsaccade-related influences on target-related activity (**Figures 1B,C**), can contribute to post-cue modulations in behavior during Posner cueing (Hafed et al., 2015). The only question that remains is whether this theoretical mechanism alone is sufficient to produce both attentional capture and IOR. In what follows, we describe a model, and experimental validation, that attempts to answer this important question. Our results do not in any way deny the concept of attention; they merely identify potential constraints on its mechanisms of action.

## Materials and Methods

**FIGURE 2:**
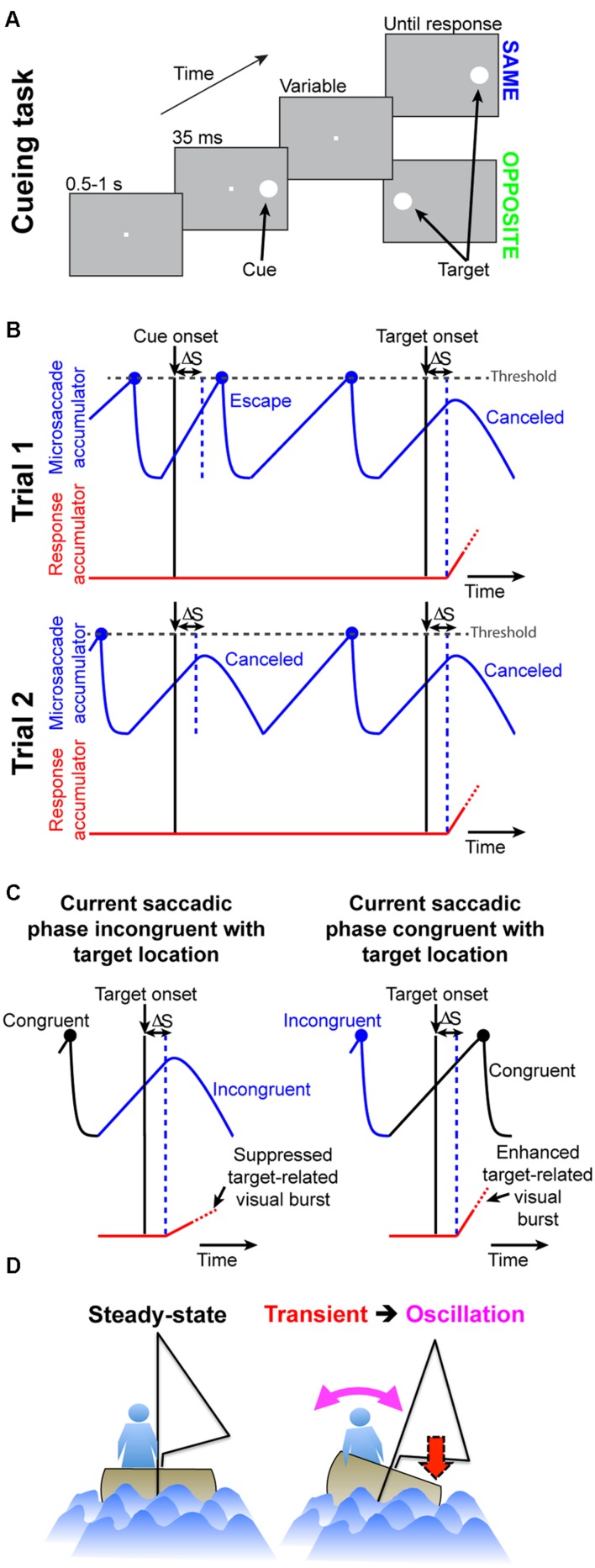
**Accounting for attentional capture and inhibition of return using repetitive microsaccades, coupled with pre-microsaccadic alteration in vision**. **(A)** Cueing task (Posner, 1980). A cue appeared followed by a target at either the “same” or “opposite” location. Two concepts of microsaccadic repetitiveness, one related to time and the other to space, are sufficient to replicate performance dynamics in this task. **(B)** In terms of time, we modeled microsaccade generation using rise-to-threshold: during fixation, microsaccades repeatedly occurred by rising to threshold (blue accumulator; Hafed and Ignashchenkova, 2013). The figure shows two example trials. When a cue/target appears, the microsaccadic rhythm is reset through “countermanding”: after a short delay, ΔS, the accumulator is slowed down to “cancel” the microsaccade. On some trials (e.g., Trial 2), the movement is successfully canceled. On other trials (e.g., Trial 1 at cue onset), the accumulator was already high enough such that it still reaches threshold; an “escape” microsaccade is executed nonetheless. In both trials, red shows the “response” accumulator, which begins to rise after target onset (this accumulator would describe manual RT’s on button press versions of the experiment; **Figure 9**). **(C)** In terms of space, microsaccades are, on average, anti-correlated in direction. For example, the right column shows a microsaccade before target onset opposite the target (blue) and the subsequent microsaccade being prepared at target onset (rising black accumulator) toward it. Movements toward a stimulus are slightly harder to cancel (e.g., right column “escape”) than movements opposite it (e.g., left column successful cancelation; Hafed and Ignashchenkova, 2013). For either case, the final response buildup rate (red) is correlated with the efficacy of microsaccade cancelation (this is a direct consequence of **Figures 1B,C**; Hafed et al., 2015). **(D)** Analogy (see Results) highlighting how our model captures cueing dynamics. A sailor’s posture continuously oscillates to maintain balance; if a gust (cue) transiently occurs, the postural oscillations become unmasked.

In Posner cueing, subjects maintain fixation while a peripheral cue and subsequent target appear (**Figure 2A**). The target could appear at either the previously cued location or opposite it. Because the eyes are never still during fixation, we wondered whether RT modulations normally observed in this task (i.e., the phenomena called “attentional capture” and “IOR”) may be a simple function of oculomotor behavior (i.e., saccades during fixation). In what follows, we describe a minimalist model that only takes into account oculomotor behavior, as well as its impact on visual sensitivity (Chen et al., 2015; i.e., *only* the phenomena of **Figure 1**), and we show how it can remarkably exhibit both attentional capture and IOR. We then describe the experiments that we used to test our model predictions.

### Computational Model

In our model, microsaccades repetitively occur, and cues reflexively reset this process (Hafed and Ignashchenkova, 2013); as a result, when post-cue targets appear, they do so at predictable phases of post-cue oculomotor behavior. At the heart of it, the model is the same as that of (Hafed and Ignashchenkova, 2013), except for the addition of a second stimulus onset (i.e., the target) after cue onset, as well as the implementation of differential microsaccade-related influence on target-related visual activity (Chen et al., 2015).

In producing the final behavior (i.e., RT to the *target* onset), our model does not use information about the locus of the previous cue or whether any top–down attentional strategy is needed. The model merely simulates a most basic microsaccadic process during fixation, which is both *repetitive in time* and *oscillatory in direction* (Hafed and Ignashchenkova, 2013). Orienting efficacy to the *target* in the model is simply a function of the *instantaneous* temporal and spatial phase of an ongoing microsaccadic plan at which the post-cue target appears (**Figures 1B,C**), and this is a direct consequence of how microsaccades influence visual sensitivity in structures critical for behavioral performance in Posner cueing (Chen et al., 2015; Hafed et al., 2015). The role of the cue is simply to reflexively reset the oculomotor system, and thus unmask systematic properties in its behavior.

The model comprises four elements (Hafed and Ignashchenkova, 2013): (1) a repetitive rise-to-threshold mechanism for generating microsaccades, (2) a reflexive resetting of microsaccades by cue/target onset, (3) an oscillatory directional pattern for microsaccades, and (4) a dynamic interaction between reflexive resetting and the direction of the movement being reset by stimulus onset. The first two elements concern the “temporal” aspects of the model (**Figure 2B**), and the last two concern the “spatial” aspects (**Figure 2C**). Again, it is important to note that in producing the final RT behavior (attentional capture or IOR), the model does not know where the previous cue was; final behavioral performance is dictated by the *instantaneous* state of microsaccade programming at the time of *target* onset (*independent* of whether a previous cue was presented or not). This is different from other models of IOR, in which explicit inhibitory signals are invoked (e.g., Satel et al., 2011).

The implementation details of our model are described next, and (as stated above) they are also similar to (Hafed and Ignashchenkova, 2013), except for the addition of a second target onset after cue onset. Moreover, the beginning of the Results section includes an analogy highlighting the simplicity of our framework, and how it can exhibit cueing dynamics (see **Figure 2D**). Finally, later in this section, we describe experimental tests for several predictions of our model; neurophysiological tests of our model predictions have been recently described (Chen et al., 2015; Hafed et al., 2015).

#### Repetitive Rise-to-Threshold Mechanism

The model utilizes a rise-to-threshold process for executing a motor output (e.g., Salinas and Stanford, 2013). In our case, we accounted for microsaccadic repetitiveness (Gaarder et al., 1966; Bosman et al., 2009) by repeatedly running this process (Hafed and Ignashchenkova, 2013).

The process consisted of a “microsaccade accumulator,” *M_microsaccade_*. Starting from a baseline of zero, the accumulator rose linearly toward threshold. The accumulator’s buildup rate was described by:

(1) $$\begin{array}{c} \frac{{dM}_{microsaccade}}{dt}=r_{B} \end{array}$$

(2) $$\begin{array}{c} r_{B}=r_{B0} \end{array}$$

For any given microsaccade, the buildup rate, *r_B_*, was a constant, *r_B0_*, that was drawn randomly at the beginning of the buildup from a gamma distribution (shape parameter *k_m_* and scale parameter *𝜃_m_*). Once *M_microsaccade_* reached threshold (1,000 arbitrary units), a microsaccade was triggered 20 ms later (Salinas and Stanford, 2013). *M_microsaccade_* decayed exponentially after reaching threshold, according to:

(3) $$\begin{array}{c} \frac{{dM}_{microsaccade}}{dt}=-\frac{M_{microsaccade}}{decay} \end{array}$$

where *decay* describes the time constant of the dropdown. When *M_microsaccade_* decayed to a value <1 arbitrary units (Hafed and Ignashchenkova, 2013), the process started anew with a new *r_B0_* for a new microsaccade. Thus, this process resulted in repetitive microsaccade generation, as occurs experimentally (Gaarder et al., 1966; Bosman et al., 2009; Hafed and Ignashchenkova, 2013). Note that the buildup rate, *r_B_*, influences inter-microsaccadic intervals. For subjects with low microsaccade frequencies, this parameter would be lower than for subjects with high frequencies. However, as we show in Section “Results,” the behavior of the model holds with different parameter values.

#### Reflexive Resetting by Cue/Target Onset

If a peripheral stimulus appears, it can be thought of as reflexively resetting the saccadic system (Hafed and Ignashchenkova, 2013). We implemented such resetting using countermanding (Hafed and Ignashchenkova, 2013; Salinas and Stanford, 2013). The stimulus acts like a “stop” signal that attempts to “cancel” the ongoing microsaccade accumulator, in order for the saccadic rhythm to restart anew (**Figure 2B**). After a brief afferent processing delay, ΔS, *M_microsaccade_* was now governed by new dynamics because *r_B_* became time varying (Salinas and Stanford, 2013):

(4) $$\begin{array}{c} \frac{{dr}_{B}}{dt}=\frac{r_{DN}-r_{B0}}{\tau } \end{array}$$

We set *r_DN_* to *–k_m_*, and *τ* was a constant that dictated how much the microsaccade accumulator was slowed down by stimulus onset. ΔS was drawn randomly from a normal distribution (mean *μ_stimulus_* and standard deviation *σ_stimulus_*).

As mentioned previously (Hafed and Ignashchenkova, 2013), the above countermanding process explains why some microsaccades can still occur after cue/target onset before the characteristic reduction in microsaccade frequency that is normally observed (Rolfs et al., 2008). If the cue/target appears when *M_microsaccade_* had risen far enough toward threshold, then the dynamics of Eq. 4 are not fast enough to prevent *M_microsaccade_* from crossing threshold. A microsaccade is thus triggered despite cancelation by stimulus onset, and this microsaccade is called an “escape” microsaccade (Hafed and Ignashchenkova, 2013). Note that as a result of this, the direction of an “escape” microsaccade provides an experimentally observable measure of the instantaneous spatial direction (see next paragraph) of the microsaccadic program that was present at target onset (i.e., the direction of the planned microsaccade at target onset). We exploited this property to test some predictions of our model (see Results).

#### An Oscillatory Direction Pattern for Microsaccades

The above model results in repetitive microsaccades (i.e., a temporal rhythm), with some microsaccades being canceled by cue/target onset and others escaping. However, microsaccades also oscillate in direction (i.e., a spatial oscillation). For example, square-waves, which are pairs of successive but oppositely directed microsaccades, are prevalent (Hafed and Clark, 2002; Bosman et al., 2009). We implemented this spatial oscillation by assigning a direction to each microsaccade. At the beginning of every trial, we picked a random direction. Any subsequent microsaccade (at the beginning of the rise of *M_microsaccade_* after the previous decay) was biased away from the previous eye movement’s direction. Its direction was drawn from a normal distribution having a mean 180° opposite the previous microsaccade direction and a standard deviation of 70° (Hafed and Ignashchenkova, 2013). This large variance allowed our model to generate both square-wave microsaccade pairs as well as single-sided (Hafed and Clark, 2002) movements, as observed experimentally. Note that our retinal-image stabilization experiments (described below) provide a possible reason for why microsaccade directions oscillate in real-life (see Results).

#### Dynamic Interaction between Reflexive Resetting and the Movement being Reset

Peripheral stimulus onset generates strong visual bursts in structures like the superior colliculus (SC), and this makes it harder to reset (i.e., countermand or cancel) a microsaccade that is being programmed toward the stimulus compared to a microsaccade that is being programmed opposite the stimulus (Hafed and Ignashchenkova, 2013). We implemented this dynamic interaction by multiplying the instantaneous accumulator rise rate (after ΔS) by a scale factor that depended on the microsaccade direction being programmed at stimulus onset: 1.02 for the same direction and 0.98 for the opposite direction. We defined “same” and “opposite” based on the horizontal component of the microsaccade relative to the horizontal location of the stimulus. The result of this interaction is that if stimulus onset happened for a microsaccade that was already being programmed toward the stimulus, visual bursts associated with the stimulus, in say, the SC made *M_microsaccade_* ever-so-slightly harder to reset than if the microsaccade was opposite. This explains why early “escape” microsaccades are highly correlated with stimulus location in our data and in previous published reports (Hafed and Ignashchenkova, 2013). The dynamic interaction term that we implemented is also consistent with large saccades, for which it was shown that the efficacy of the countermanding process depended on the properties of the saccade being countermanded (Montagnini and Chelazzi, 2009). Finally, please note that this scaling term is *only* necessary in the current model because the model does not implement a spatial map of locations. If we replicate this model with a spatial map instead (data not shown), then the term becomes automatically implemented by the locus of the peripheral visual burst.

In the current model, we assumed that if a microsaccade was successfully canceled by the peripheral stimulus onset, the next microsaccade after the reset was biased opposite the stimulus (with a similar angular standard deviation of 70°). We used this rule for the first microsaccade after successful cancelation only to simplify the model, but it was consistent with our data. In fact, we also ran an identical version of the original model of (Hafed and Ignashchenkova, 2013; i.e., without this rule) but adapted to include both cue and target onsets as in Posner cueing experiments, and we could still replicate the results of the current paper (data not shown). Here, we elected to simplify the model even further to emphasize that Posner cueing dynamics can be replicated with the simplest possible model that maintains the above four key elements.

The above model accounted for microsaccadic modulations (**Figure 1A**). To model the final behavioral output (whether saccade or manual button-press RT), we assumed that target onset releases a response accumulator, *M_response_* (e.g., **Figures 2B,C**), whose slope is dictated by the strength of target-related visual bursts (**Figures 1C and 2C**; Hafed et al., 2015). Thus, after the afferent processing delay, ΔS, the microsaccade accumulator was attenuated as usual after stimulus onset (e.g., Eq. 4), and it was stopped after either a successful cancelation or an “escape” microsaccade. A second “response” accumulator started rising after ΔS. This accumulator represents the recruitment of populations of neurons (other than those needed for microsaccades) in, say, SC in order to initiate the final eye movement (Munoz and Wurtz, 1995) or button decision. The accumulator was identical to Eq. 1. In this case, *r_B0_* was drawn from a normal distribution (mean *μ_response_*; standard deviation *σ_response_*). To simulate the influences of microsaccades on behavioral and neuronal responses, independent of prior cueing (Hafed, 2013; Chen et al., 2015; **Figure 1C**), we modulated the sensitivity of the response accumulator by the current plan of the microsaccadic system at which the target appeared. This aspect of the model *directly* simulates the differential pre-microsaccadic changes in vision that take place around the time of these small eye movements (Chen et al., 2015; **Figures 1B,C**). Specifically, if the microsaccade accumulator at target onset was rising for a microsaccade in the direction of the appearing target, then this meant that the target appeared congruent with the spatial direction of the microsaccadic plan. In this case, the randomly drawn response accumulator value *r_B0_* was scaled up by a factor of 1.25, modeling an enhanced visual response to the target in structures like the SC (Chen et al., 2015; **Figure 1C**). If, on the other hand, the microsaccade accumulator was rising for a movement opposite the target location when the target appeared, then the target appeared in conflict with current spatial direction of microsaccade plans. In this case, the target was less effective in driving the final decision, and we scaled the response accumulator *r_B0_* by a factor of 0.7, modeling a suppressed visual response (Chen et al., 2015; **Figure 1C**). If the microsaccade accumulator was declining at target onset, no modulation of *r_B0_* was invoked, because experimental evidence reveals no direction-dependent differential effect in the post-microsaccadic interval (**Figure 1B**). It is important to note here that these modulations in *r_B0_* are directly consistent with neurophysiological evidence that SC target-related activity is strong for fast RT’s and weaker for IOR (Dorris et al., 2002; Fecteau et al., 2004; Fecteau and Munoz, 2005; Hafed et al., 2015), but they occur in our model *only* as a function of microsaccades and *independent* of prior cueing. Moreover, such modulations appear on initial target-related visual bursts, which explains why the SC (a saccade structure) is causally involved in IOR even when manual responses are used (Sapir et al., 1999). Finally, whether with saccades or with buttons, such SC visual bursts (target-related activity) are a correlate of the slope of rise-to-threshold processes (Carpenter and Williams, 1995; Boehnke and Munoz, 2008; Hafed et al., 2015). Therefore, all of the above suggests that a strong *prediction* of our model is that “strong” and “weak” neural activity in response to target onset would be temporally synchronized with, and significantly modulated by, microsaccades (and independently of any cueing). We have indeed recently confirmed this prediction neurophysiologically (Chen et al., 2015). In the present paper, we have also further confirmed this model prediction using behavioral experiments (see below).

We re-iterate that when the target appears, our model dictates final RT (i.e., the slope of the response accumulator) with no knowledge about the previous cue location or the “desired” locus of peripheral covert attention. It only generates behavior based on whether the target appears in or out of spatial phase with the current, *instantaneous* microsaccadic plan (see analogy in **Figure 2D**). This suggests that in dictating final behavioral performance, our model is agnostic of the particular implementation of the *M_microsaccade_* model above; *any* simulation that will replicate well-known reflexive microsaccadic behavior during Posner cueing will also generate attentional capture and IOR *if* the peri-microsaccadic modulations of the response accumulator that we mentioned here are implemented. Thus, from a theoretical perspective, our model (see Results) is *sufficient* to generate behavioral modulations in Posner cueing. According to the model, the reason different cue-onset-to-target-onset asynchronies (CTOA’s) result in different performance in Posner cueing is a simple result of different phases of post-cue microsaccadic rhythms at which targets appear (Results).

**FIGURE 3:**
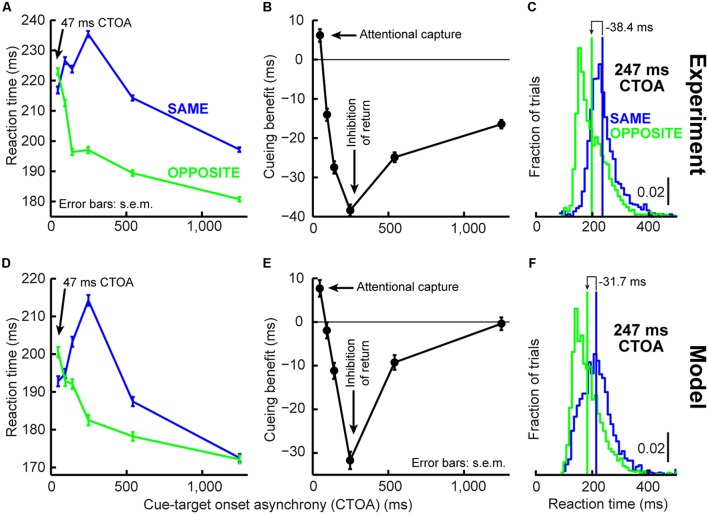
**Orienting dynamics in experiment and model. (A)** Saccade reaction time (RT) in the cueing task of **Figure 2A** as a function of CTOA for “same” and “opposite” trials. RT was faster for “same” at 47 ms CTOA but slower later. **(B)** Cueing benefit defined as the RT difference between “opposite” and “same,” highlighting the benefits and costs in **(A)**. **(C)** RT distribution for the CTOA with maximal IOR. “Opposite” was faster on average but had similar variability range as “same.” Please also see **Figure 4B** (top) and Supplementary Figure S1 for RT distributions from all other CTOA’s. **(D–F)** Model results capturing the dynamics of the experimental data in **(A–C)**. Subsequent figures also show individual subject results and model simulations with reduced trial numbers. All error bars denote SEM. Each data point has *N* ∼2,000–3,000 trials.

**FIGURE 4:**
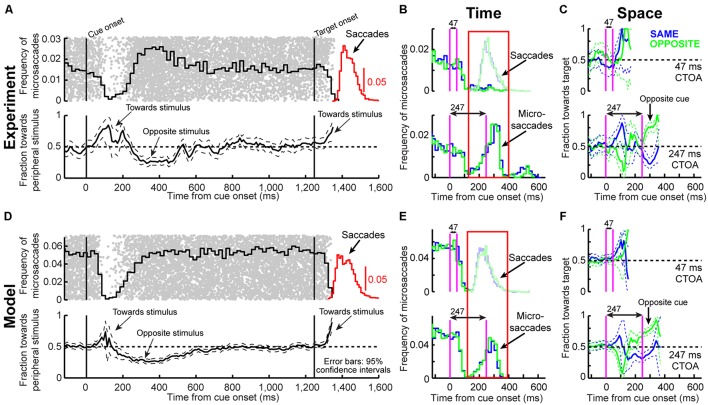
**Microsaccade dynamics in the cueing task (experiment and model). (A)** Microsaccade frequency (top) and direction (bottom) as a function of time for 1,247 ms CTOA’s (>4,000 trials). Top plots the fraction of trials containing microsaccades. Bottom plots the fraction of microsaccades directed toward the peripheral stimulus. Red indicates RT for large saccades (note specific scale bar). Microsaccades exhibited well-known modulations. Gray dots are rasters of microsaccade onset times across trials. **(B)** Microsaccade frequency in two CTOA’s (top and bottom). Colors refer to the location of the target relative to the cue (blue for “same”). The faint histograms show saccade RT’s with similar color coding. Longer CTOA’s (bottom) exhibited microsaccades at the time at which large saccades would have occurred if fixation was not enforced (compare to the saccade RT’s in the short CTOA’s – red rectangle). Magenta lines indicate cue/target onset. **(C)** Microsaccade directions in the CTOA’s of **(B)**. When there was sufficient time between cue and target, microsaccades were initially biased toward the cue (thus opposite the target for the green curves in which the cue was opposite the target location). For 247 ms, most microsaccades near target onset were toward the target in the opposite condition because they had flipped from being toward the cue earlier. **(D–F)** Model simulations from the scheme of **Figure 2** capturing all the salient features of the data. All error bars denote 95% confidence intervals. Each experimental condition has *N* ∼2,000–3,000 trials; simulations: 2,000 trials.

**FIGURE 5:**
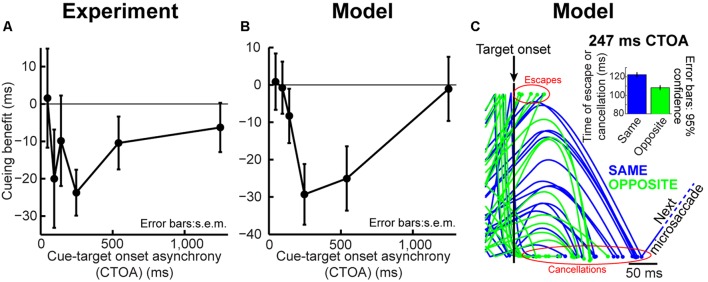
**Dissecting performance dynamics in the cueing task. (A)** Cueing benefit (as defined in **Figure 3**) computed for the first microsaccade RT after target onset. Like larger saccades, these “escapes” exhibited similar dynamics after cue onset, with maximal IOR-like behavior at ∼247 ms. Error bars denote SEM. **(B)** Similar analysis for our model data. We ran the model microsaccade generator for each CTOA for 2,000 trials, and we measured first microsaccade RT. The first microsaccade exhibited cueing dynamics. Attentional capture at the shortest CTOA was less obvious (even in the real data) because of low microsaccade frequency at this CTOA (see **Figure 6**). Thus, even when analyzing the distribution of microsaccades occurring after target onset, their RT’s exhibited dynamics similar to **Figure 3**. **(C)** Twenty randomly chosen model trials from the “same” (blue) and “opposite” (green) conditions showing target influence at 247 ms CTOA. Most microsaccades were canceled by target onset, as expected (slowing of accumulator processes toward zero). However, because most microsaccades in the “opposite” condition were toward the target (**Figures 4C,F**), both the cancelation and escape mechanisms showed different temporal dynamics from the “same” condition, in which most microsaccades were opposite the target. The inset shows the combined latency of all canceled and escape time points at this CTOA, showing IOR (i.e., opposite faster than same). Inset error bars denote 95% confidence intervals.

**FIGURE 6:**
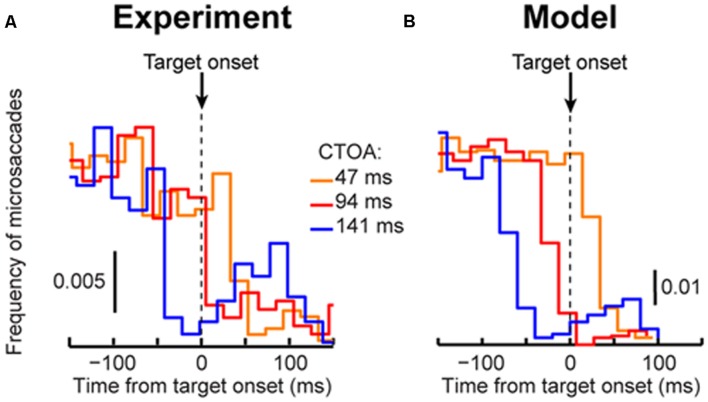
**Our model can account for cueing dynamics even if microsaccades are rare. (A)** Microsaccade frequency around target onset for the shortest three CTOA’s in “same” trials. Virtually identical curves were obtained from “opposite” trials. Each curve shows data from >2,000 trials. The timing between target and cue onset caused modulations in microsaccade frequency as a function of CTOA. For example, there were extremely few microsaccades near target onset in 94 ms CTOA trials even though RT in these trials was markedly different from RT in other CTOA’s (see **Figure 3**). **(B)** Model microsaccades showed similar modulations (see **Figure 4** for other CTOA’s). In this case, we ran the model for 2,000 trials per CTOA. Thus, the model captured large changes in RT across CTOA (**Figure 3**) even when microsaccades around target onset were rare. The rarity and rebound were a function of the resetting of microsaccadic rhythms (Hafed and Ignashchenkova, 2013). Importantly, microsaccade sparseness did not prevent replicating individual subject data (see **Figure 7**).

**FIGURE 7:**
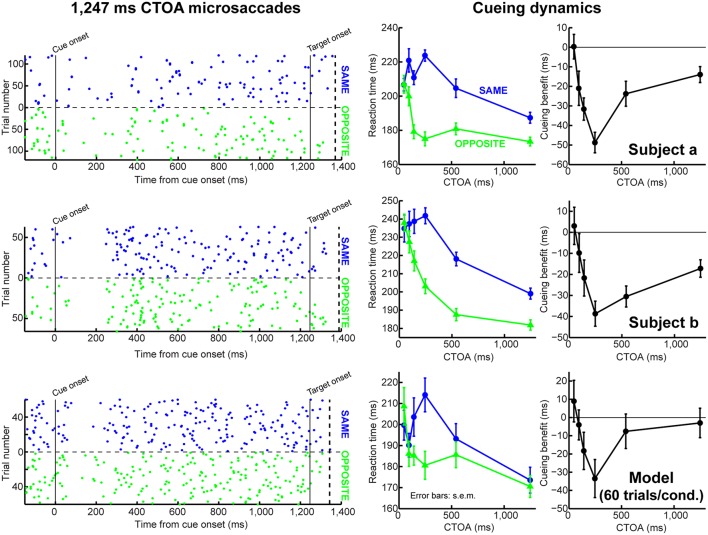
**Accounting for behavioral dynamics even with few microsaccades.** In this figure, we show data from individual subjects (first two rows) and from the model when it was run with a small number of trials (third row). The first column shows microsaccade onset times (as dot rasters) for 1,247 ms CTOA’s. Each dot indicates a microsaccade onset, and each row corresponds to a single trial. The trials were sorted according to the target being at the same (blue) or opposite (green) location as the cue. The right two columns show behavioral dynamics. In the third row, we ran the model with only 60 trials per condition, which is the smallest number of repetitions that we got from one of our subjects. Thus, even when simulated as an individual subject, the model could replicate behavioral dynamics. Importantly, microsaccades did not happen particularly frequently in this simulation (dot rasters), but this was still sufficient to generate realistic dynamics (right two columns). The dashed vertical line in the left column graphs indicates the earliest RT observed for each subject/model. Also, note that different individual subjects had different intrinsic microsaccade frequencies (compare dot rasters from subjects a and b). **Figure 8** demonstrates how our model can also simulate such inter-subject variability. All error bars denote SEM.

We simulated 2,000 trials per condition, which is similar to our experimental trial numbers (see below). We also simulated the model as an “individual subject” by reducing the number of trials per condition and with different dynamics. **Table 1** summarizes the model parameters that were not explicitly mentioned above. In obtaining such model parameters, we did not perform explicit parameter optimization routines; instead, we were guided by earlier physiologically inspired work (Hafed and Ignashchenkova, 2013; Salinas and Stanford, 2013), as well as by the behavioral data from the experimental portions of this study (**Figures 3–7**). For our microsaccade-dependent scaling of response accumulator slope according to microsaccade direction, we specifically used gain enhancement and suppression factors similar to those we recently identified physiologically (Chen et al., 2015). We should also note that in Section “Results,” we additionally tested the robustness of our model by performing large parameter sweeps and investigating how these sweeps altered the dynamics of the model.

**Table 1:** Model parameters that were not already mentioned inline in Section “Materials and Methods.”

Parameter	Value
ΔS	Normal distribution: Mean (*μ_stimulus_*) 30, standard deviation (*σ_stimulus_*) 12
Buildup rate for microsaccade (*r_B_*)	Gamma distribution: Shape parameter (*k_m_*) 1.6, scale parameter (𝜃_m_) 2.66
Buildup rate for final RT (*r_B_*)	Normal distribution: Mean (*μ_response_*) 8 for CTOA’s less than 541 ms; 9 and 9.5 for 541 ms and 1,247 ms CTOA’s, respectively; standard deviation (*σ_response_*) 2
*decay* (Eq. 3)	7
*τ* (Eq. 4)	37

*Note that if parameters drawn from a normal distribution turned out to be negative, they were resampled until positive values were obtained. This ensured non-negative processing delays or buildup rates in the model.*

### Behavioral Tasks

All experiments were approved by ethics committees: at Tuebingen University for human experiments, and at the regional governmental offices of the city of Tuebingen for monkey experiments. All human subjects gave written informed consent in accordance with the Declaration of Helsinki.

#### Cueing Task

We first implemented the Posner cueing task to experimentally replicate attentional capture and IOR, and to validate our model results. Human subjects viewed a display consisting of a gray background and a central fixation spot (see **Figure 2A**). After 500–1,000 ms of fixation, a cue (1° diameter white circle of similar luminance to the spot) appeared for ∼35 ms 5° to the right/left of fixation. After one of six possible CTOA’s (47, 94, 141, 247, 541, or 1,247 ms), an identical circle appeared at the previously cued location (“same”) or opposite it. The fixation spot was removed simultaneously, and subjects oriented to the target with a saccade as fast as possible. Target location was equally likely to be at the cued or uncued location.

The experiments were conducted in a dark room with subjects seated 57 cm in front of a CRT monitor (41 pixels/°; 85 Hz). The fixation spot (7.3′ × 7.3′) was white (97.3 cd/m^2^ luminance), and background luminance was 20.5 cd/m^2^. We tracked eye movements using a high-speed camera (EyeLink 1000, 1 kHz sampling). We fixed subjects’ heads at five points using a custom-made fixation device (Hafed, 2013).

Subjects participated in 3–5 1-h sessions, and we collected 480–600 trials per session. We analyzed data from 22 subjects (12 male), aged 23–37 years, resulting in ∼2,000–3,000 trials per experimental condition (∼60–200 trials/condition/subject).

#### Button and Saccade Reaction Time Tasks

A prediction of our model (**Figure 2C**) is that visual sensitivity to a target is modulated by the *instantaneous* spatial and temporal phase of microsaccadic programming rhythms at which the target appears (and *independently of prior cueing*): if a target appears in a direction spatially congruent with the current microsaccadic plan, sensitivity to the target is higher than if the same target appears in a direction incongruent with the current microsaccadic plan (**Figure 1B**). If this model prediction (and supporting neurophysiology; Chen et al., 2015) is valid, then similar RT changes to those seen in the cueing task above should also appear in a task without cueing, but rather as a function of the time and direction of microsaccades around target onset. Moreover, such effects should also appear independently of response modality, whether RT is measured with saccadic or manual button presses. We thus ran saccadic and manual button-press RT tasks without cueing, and we analyzed the effects of microsaccades. The saccade tasks were run on two rhesus macaque monkeys (see below), and we also confirmed the monkey results with one human subject (whose data we are not showing for brevity). The button task was run on eight human subjects (two males).

The human saccade task required the subject to fixate a central spot for 0.25–5 s. The spot was then removed, and the same stimulus as our cue/target above appeared 5° to the right/left. In the button version of the task, the fixation spot remained on during target presentation, and subjects maintained fixation but pressed a button as fast as possible. The target was visible until response time. The monkey saccade task was similar except that the initial fixation interval was shorter (700–2,000 ms) and the target was a vertical sine-wave gabor grating of high (80%) contrast, different spatial frequencies (five possibilities in the range of 0.56–11.11 cpd) interleaved across trials, 1° radius, and presented at 3.5° eccentricity to the right/left. Grating phase was randomized on every trial. Animal preparation, and the laboratory setup for the monkeys was described earlier (Chen and Hafed, 2013; Hafed and Ignashchenkova, 2013).

We collected 7,843 trials from monkey P, 8,713 trials from monkey N, and 776 trials from the human in the saccade task. We collected 3,628 trials from the button task.

#### Retinal-Image Stabilization Task

Our model utilizes microsaccadic repetitiveness, coupled with peri-microsaccadic modulations of visual sensitivity, to generate both attentional capture and IOR (see Results). To understand why microsaccades are governed by an oscillatory rhythm (Gaarder et al., 1966; Bosman et al., 2009; Hafed and Ignashchenkova, 2013) in the first place, we hypothesized that such rhythmicity is a function of instantaneous foveal motor error. That is, we reasoned that microsaccades primarily correct for foveal motor error (Guerrasio et al., 2010; Ko et al., 2010), and that post-cue microsaccadic oscillations (Hafed and Ignashchenkova, 2013) reflect disruptive influences of the cue on the oculomotor system (see Results). If this were the case, then experimentally controlling instantaneous foveal motor error should alter post-cue microsaccadic oscillations. We tested for such alteration by applying retinal-image stabilization and manipulating instantaneous foveal motor error despite continuous fixational eye movements. We employed the same two adult, male rhesus macaque monkeys mentioned above. These monkeys (aged 7 years) were ideal for using retinal-image stabilization because they were implanted with scleral search coils (Fuchs and Robinson, 1966; Judge et al., 1980); retinal-image stabilization in humans would not have been feasible with our video-based eye tracking system due to large uncertainty in absolute eye position using pupil-tracking video approaches.

The monkeys sat 45 cm in front of a CRT monitor (22 pixels/°; 120 Hz). To achieve retinal-image stabilization, we sampled eye position in real-time and updated the display at the fastest possible rate (i.e., every frame refresh; ∼8 ms). This rate was only limited by the maximum display rate, since our experimental system (Chen and Hafed, 2013; Hafed and Ignashchenkova, 2013) can achieve 1 ms. However, our sampling interval is similar to that used successfully before for retinal-image stabilization experiments (Rucci et al., 2007; Santini et al., 2007; Poletti et al., 2013). Moreover, since the majority of time during fixation is microsaccade-free fixation, the system was effective in reducing retinal image motion as we intended to do.

The specific task that we used was as follows. The monkeys fixated a central spot (8.5′ × 8.5′; 72 cd/m^2^) presented over a gray background (21 cd/m^2^). After a fixation interval (400–900 ms), control trials consisted of a cue appearing at 5° horizontally or vertically. The cue was a disk that was white at the center and gradually approached background luminance according to a Gaussian profile with 1° standard deviation. The cue remained on for 750–1,250 ms, after which the fixation spot disappeared instructing the monkeys to foveate the cue. The monkeys were rewarded for maintaining gaze within 1° from the fixation spot and then bringing gaze within 2° from the cue location at trial end. On randomly interleaved trials, retinal-image stabilization was applied. After the fixation interval, the fixation spot was translated in register with the monkeys’ eye position. This stabilization lasted for 100–550 ms, after which the cue appeared (also in a stabilized manner). The cue and fixation spot remained stabilized for the same interval as in the control condition, after which the peripheral stimulus froze and the fixation spot was removed. The monkeys then foveated the stimulus with a 5° saccade. During stabilization, there was no constraint on eye position, since the foveal stimulus was always moved with gaze. Success at the end of the trial only depended on bringing the eye within 2° from the now-stationary cue location. In yet additional interleaved trials, we applied retinal-image stabilization but now forcing the fixation spot to remain ∼2.7′ to the right or left of current gaze position. Thus, if the cue was to the right and the fixation spot was stabilized 2.7′ to the left of current gaze, then this was a condition in which foveal motor error was opposite the cue direction. If the cue was to the left, then foveal motor error was toward the cue.

We calibrated eye data for online stimulus updates using a 19-point grid. We collected multiple raw tracker values from each location and then fitted the measurements with polynomials of the form:

(5) $$\begin{array}{c} {\begin{array}{c} epos \end{array}}_{h_{calibrated}}=a\times {{epos}_{h}}_{raw}+b\times {{epos}_{v}}_{raw}+c\times {{epos}_{h}}_{raw}\times {{epos}_{v}}_{raw}+d\times {epos}_{h_{raw}}^{2}+e\times {epos}_{v_{raw}}^{2} \end{array}$$

where *epos_h_* and *epos_v_* are horizontal and vertical eye position, respectively, and “raw” and “calibrated” indicate the values of such eye position before/after calibration. Calibration consisted of finding the parameters *a*, *b*, *c*, and so on that fit the calibration measurements (Stampe, 1993). We applied a similar procedure for vertical eye position. We then took these parameters and used them in real-time to update stimulus display coordinates according to eye position. Since search coil systems can drift, we applied an offset correction at the beginning of every trial when the monkey was properly fixating. Extensive experiments make us confident that eye tracker drift is limited to offsets and not gain changes. Finally, we used high-speed Ethernet connections for display updates, and we checked whether we missed frames due to communication delays. Using our real-time system, we never missed any display update (∼millions of updates).

We analyzed 13,973 control trials and 13,908 stabilization trials. Out of the latter, 8,705 were full stabilization trials, 2,596 were with the fixation spot forced 2.7′ toward the cue location, and 2,607 were with the fixation spot opposite the cue location.

### Data Analysis

We detected saccades/microsaccades in all experiments using eye velocity/acceleration criteria (Hafed et al., 2009). We inspected all data to correct false alarms/misses.

In the cueing task, we classified as a correct orienting saccade a movement >3.5° toward the target. We analyzed microsaccades only up to large saccade execution, which constituted trial end. RT was measured as the time of large saccade onset relative to target onset. To obtain a “cueing benefit,” we plotted the difference between RT on “opposite” and “same” trials. A positive difference indicated that “same” had faster RT than “opposite” (a cueing benefit; Fecteau and Munoz, 2005).

In the saccade reaction time task, we classified as a correct saccade a movement within 1–1.5° from target center. Moreover, RT was measured in this task (and in the button version of it) similarly to how we measured it for the cueing task.

To estimate microsaccade frequency, we plotted histograms aligned on cue/target onset using 20 ms bin widths, and we normalized the histograms by the number of trials. To estimate microsaccade direction oscillation time courses in the human experiments, we used an analysis described recently (Hafed and Ignashchenkova, 2013). We analyzed model data identically to experimental data.

For the button-press experiment, we analyzed RT as a function of “escape” microsaccade direction. We took only trials with a single microsaccade <100 ms after target onset (i.e., an “escape” microsaccade) and no other microsaccades either <50 ms before target onset or >100 ms after target onset (and until the RT). We then asked whether RT was faster or slower if the target appeared congruent with that single “escape” microsaccade direction or not. We also compared these data to data in which the single microsaccade occurred 50–150 ms before target onset. We used a similar procedure for the saccade version of the task, except that we narrowed the “escape” interval to <50 ms after target onset.

For the retinal-image stabilization experiments, we binned microsaccade directions into four quadrants: within ±45° from the cued direction, the opposite direction, or the two “neither” directions. For each time bin (same bin width and step as in the human experiments above), we plotted the fraction of all microsaccades occurring within the time bin that were either toward the cue, opposite it, or to neither direction. With this approach, if microsaccades were equally distributed in direction, each of these fractions would be at 0.25 (chance level).

In several analyses, we pooled data across subjects, which was justified for several reasons. First, all of our effects, whether on RT or microsaccade frequency/direction, are robust and observed across different laboratories and animal models (humans and monkeys). Second, we repeated analyses on individual subjects, and our conclusions were unaltered (see Results). Finally, even if individual subjects have different microsaccade frequencies, this does not alter our conclusions. The space/time concepts of our model hold with different microsaccade rates, with the differences being in the behavioral dynamics. We confirmed this both experimentally and with model simulations.

## Results

### Capturing Attentional Capture and Inhibition of Return using Microsaccades

We implemented the Posner cueing task (Posner, 1980; **Figure 2A**, see Materials and Methods). Humans fixated a spot while a brief cue appeared. After a CTOA, the spot disappeared and a target appeared at the cued or opposite location. For short CTOA’s, subjects oriented to the target faster if it appeared at the cued location than if it appeared at the opposite location (**Figure 3A**, 47 ms CTOA, *p* = 1.1^∗^10^–4^, two-sided *t*-test between same and opposite). This phenomenon (called “attentional capture”; Jonides, 1981; Egeth and Yantis, 1997; Fecteau and Munoz, 2005) was short-lived, however, because subjects got much worse later: by 247 ms CTOA, RT was 235 ms at the cued location but only 197 ms opposite (**Figure 3A**, 247 ms CTOA, *p* = 1.7^∗^10^–141^, two-sided *t*-test between same and opposite). Thus, our subjects replicated classic attentional capture and IOR, and with similar dynamics (**Figures 3B,C**; also see Supplementary Figure S1).

We tested whether such dynamics can, from a theoretical perspective, be replicated (**Figures 3D–F**) by a model that only takes the concepts of **Figure 1** into account, without any other assumptions about “attention” or “IOR.” The concept of the model is as follows (**Figure 2**; implementation details are provided in Section “Materials and Methods”): a microsaccadic process (Hafed et al., 2009) repeatedly rose toward threshold to trigger a movement. Once the movement was executed, the process rose again to maintain a certain rhythm (**Figure 2B**), which also directionally oscillated (Hafed and Clark, 2002; Engbert and Kliegl, 2003; Hafed et al., 2011; Hafed, 2013; Hafed and Ignashchenkova, 2013; **Figure 2C**, see Materials and Methods). Rhythm maintenance was ensured in the model by microsaccade accumulator buildup rate (**Figure 2B**), which was drawn for every microsaccade from a stochastic distribution having a dominant range (see Materials and Methods); microsaccade times were thus variable, but predominantly rhythmic (Bosman et al., 2009; Hafed and Ignashchenkova, 2013). Moreover, a dominant directional oscillation was ensured by a likelihood that an upcoming microsaccade was opposite the most recent one (see Materials and Methods). If a cue/target were to now appear, the rhythm was reset after a short delay, ΔS (Hafed and Ignashchenkova, 2013; **Figures 2B,C**). Subsequent targets then appeared at distinct phases (both temporal and spatial) of the reset rhythm, resulting in predictable behavioral modulations with different CTOA’s.

To understand how this simple model was *sufficient* to generate cueing dynamics, consider first an analogy. Imagine a sailboat in wavy waters (**Figure 2D**). While the sailor repeatedly oscillates to balance herself, measuring her posture at random times yields, on average, a balanced upright position (**Figure 2D**, steady-state). If, however, a wind gust transiently fills the sail, the sailor will initially tip in the gust’s direction. This effect will soon reverse because she will lean opposite to rebalance. Thus, post-gust, the sailor’s postural oscillations would be similar to her earlier oscillations (all aimed at balancing herself), but they would be reset, and thus *unmasked*, by the gust. Our model (**Figure 2**, see Materials and Methods) implemented this concept in the context of the saccadic system (the sailor), without any top–down peripheral covert attentional processing: microsaccades were continuously oscillating to optimize eye position, and these oscillations were reset (and thus unmasked) by stimuli. The model was sufficient to replicate the dynamics of our data (**Figures 3D–F**), even on an individual-subject basis (shown later). Our approach, while simple, represents a stark contrast from existing theories of peripheral covert attention. This approach demonstrates that (at least from a theoretical perspective) repetitive microsaccade generation, coupled with peri-microsaccadic changes in visual sensitivity (see Materials and Methods; Chen et al., 2015; **Figures 1B,C**), can be sufficient to cause behavioral modulations that are observed in the Posner cueing task.

We next detail how our model accomplishes its performance, how it generalizes to manual responses, and how it means that performance modulations to a target can even occur without any cues at all. We then end by showing that microsaccadic repetitiveness itself reflects oculomotor control over foveal motor error.

### Time and Space in Microsaccade Generation Dictate whether Attentional Capture or Inhibition of Return Are Observed

From a theoretical perspective, microsaccades can account for cueing dynamics because of the influence of stimulus onsets on such repetitiveness. Consistent with previous results (Hafed and Clark, 2002; Engbert and Kliegl, 2003; Galfano et al., 2004; Betta et al., 2007; Hafed et al., 2011; Hafed and Ignashchenkova, 2013), cue onset altered both microsaccade frequency (**Figure 4A**, top) and direction (**Figure 4A**, bottom) in a machine-like manner, and microsaccades were biased away from the cue at times of maximal IOR (i.e., ∼247 ms). Prior work (e.g., Hafed and Clark, 2002; Galfano et al., 2004; Betta et al., 2007) has assumed that such correlation arose because microsaccades trailed peripheral covert attentional shifts. By acknowledging that microsaccades are mechanistically similar to saccades (Hafed et al., 2009), are associated with peri-microsaccadic changes in vision that can themselves alter behavioral and neuronal performance (Zuber and Stark, 1966; Herrington et al., 2009; Hafed and Krauzlis, 2010; Hafed, 2013; Chen et al., 2015; Hafed et al., 2015), and are governed by similar rhythmicity (Gaarder et al., 1966; Bosman et al., 2009; Hafed and Ignashchenkova, 2013), we were now able to understand how microsaccade/saccade generation itself could be *sufficient* (at least theoretically) to account for cueing dynamics.

According to the model, two concepts, one concerned with time (**Figure 4B**) and the other with space (**Figure 4C**), can be enough to account for cueing dynamics. In terms of time, microsaccade frequency abruptly “stops” and then recovers (e.g., **Figure 4A**, top). This stop represents a cue-induced temporal-frequency “phase resetting” (Hafed and Ignashchenkova, 2013), and we implemented it through countermanding (Hafed and Ignashchenkova, 2013; Salinas and Stanford, 2013; e.g., **Figure 2**). The implication of this reflexive resetting is that during fixation, microsaccades will still occur after the resetting event such that the saccadic system’s temporal structure (Gaarder et al., 1966; Bosman et al., 2009; Hafed and Ignashchenkova, 2013) is still maintained. For example, with 247 ms CTOA’s, a population of tiny microsaccades occurred at roughly the same time after cue onset as the 5° targeting saccades of the shorter 47 ms CTOA trials when fixation was released (**Figure 4B**, red rectangle): the saccadic system still generated motor outputs after cues, but the movements were small with a persistent foveal stimulus instead of large when fixation was released. As a result of this, and given saccade/microsaccade repetitiveness (Gaarder et al., 1966; Bosman et al., 2009; Drewes and VanRullen, 2011; Hafed and Ignashchenkova, 2013), final RT clearly depended on the previous microsaccadic temporal structure.

The second concept has to do with space. On average, microsaccades in our model oscillate in direction (Hafed and Clark, 2002; Engbert and Kliegl, 2003; Bosman et al., 2009; Hafed et al., 2011; Hafed, 2013; Hafed and Ignashchenkova, 2013), and such oscillations are also cue-reset: early microsaccades “escaping” the temporal-frequency phase resetting are more likely to be toward the cue than opposite, resulting in coherent post-cue direction oscillations (**Figure 4A**, bottom). This phenomenon is consistent with earlier evidence of an interaction between “escape” movements and countermanding (Montagnini and Chelazzi, 2009; Hafed and Ignashchenkova, 2013), and its likely neuronal substrates are reflexive, cue-induced SC visual bursts (Hafed and Ignashchenkova, 2013). In the model, the dynamics of this phenomenon (i.e., its speed and duration) were dictated by the efficacy of cue-/target-related sensory processing (ΔS), as well as the efficacy with which sensory inputs countermanded the microsaccadic buildup accumulator (Δ, see Materials and Methods). Intuitively, these dynamics are consistent with the sailor analogy (**Figure 2D**): if the sailor was already leaning in the direction of an upcoming gust, the gust is more likely to tip her in its direction than if she was leaning opposite. This means that, relative to the final target position, “same” and “opposite” cue onsets in our task caused counterphase direction oscillations (**Figure 4A**, bottom and **Figure 4C**). When the target later appeared, it could do so when the saccadic system was either preparing to move in the direction of the cue or opposite it (**Figure 4C**), which ultimately affected final RT. Interestingly, such an influence of microsaccadic directional preparedness on final RT (e.g., **Figure 2C**) predicts a far-reaching impact of microsaccades on peripheral target representations (which can be multiple orders of magnitude farther than the microsaccade endpoints). This prediction was recently supported neurophysiologically (Hafed and Krauzlis, 2010; Ghitani et al., 2014; Chen et al., 2015; Hafed et al., 2015) and also behaviorally (Hafed, 2013).

Using both time and space, we can now understand why 247 ms CTOA showed the strongest IOR (**Figure 3**). For 247 ms “same” trials, the target appeared at a spatial phase in which the saccadic system had already flipped away from preparing movements toward the cue to preparing ones opposite (**Figure 4C**, 247 ms CTOA, blue), and the timing of this flip was dictated in the model by the dominant microsaccadic rhythm speed (or accumulator buildup rate in **Figure 2**). According to our model (**Figures 4D–F**) and the sailor analogy (**Figure 2D**), target onset in this case (“same” trials) was tantamount to a second wind gust occurring in a direction opposite the sailor’s current tilt; it was less effective in pushing the sailor than if the sailor had been tilting in the gust’s direction. Thus, our model not only replicated cueing dynamics (**Figure 3**), but it also did so by accounting for the fine-scale oculomotor modulations in the task (**Figure 4**), and using very simple active vision concepts (**Figure 1**).

A strong prediction of our framework is that cueing-like dynamics should manifest themselves not only in final RT’s (**Figure 3**), but also in the “escape” microsaccades occurring after target onset (but before the final RT itself); this is so because RT in our framework reflects resetting of an entire ongoing saccadic rhythm, and “escape” microsaccades are part of this rhythm. We thus analyzed first microsaccade RT’s rather than final RT’s (**Figure 5A**). Consistent with our model prediction (**Figure 5B**), first microsaccades exhibited cueing time courses like the final behavioral responses. When inspecting the microsaccadic accumulator process of the model (**Figure 5C**), we could understand why: target onset canceled most microsaccades, as expected, but there was a difference in the timing of “canceled” and “escape” microsaccades between same and opposite conditions; at 247 ms, most microsaccades in the “opposite” condition were in spatial phase with target location, whereas most microsaccades were directionally incongruent with the target in the “same” condition (**Figures 4C,F**, 247 ms CTOA), resulting in maximally different temporal dynamics. Thus, according to the model, IOR may simply be a result of the state of *current* saccadic (or sailor) phase at target (gust) onset, independent of the prior cue.

### Microsaccades Have a Measurable Influence Even If They Are Rare

It may be argued that microsaccades occur too infrequently in cueing tasks to be of much importance. However, we observed that our model exhibited an interesting emergent property; it explained how microsaccades can sometimes be quite infrequent but still influential. Consider, for example, the first three CTOA’s, which resulted in highly different RT’s experimentally (**Figures 3A,D**). Target onset occurred at different phases relative to the previous cue-resetting event in these (and other) CTOA’s. As a result, these CTOA’s caused marked microsaccade frequency modulations, such that 94 and 141 ms CTOA’s had rare microsaccades at target onset (**Figure 6A**). Despite these modulations, which our model captured (**Figure 6B**), our model still exhibited markedly different RT’s at these three CTOA’s, as in the experiments (**Figure 3D**). In fact, when we ran the model with only 60 trials per condition, to simulate an individual subject, the model still modulated RT and exhibited IOR (**Figure 7**), even though microsaccades were rare. Thus, microsaccade sparseness does not necessarily indicate irrelevance, as it is the entire cyclic process of the saccadic system that matters.

### Variability of Microsaccades Correlates with Variability in Cueing Effects

**FIGURE 8:**
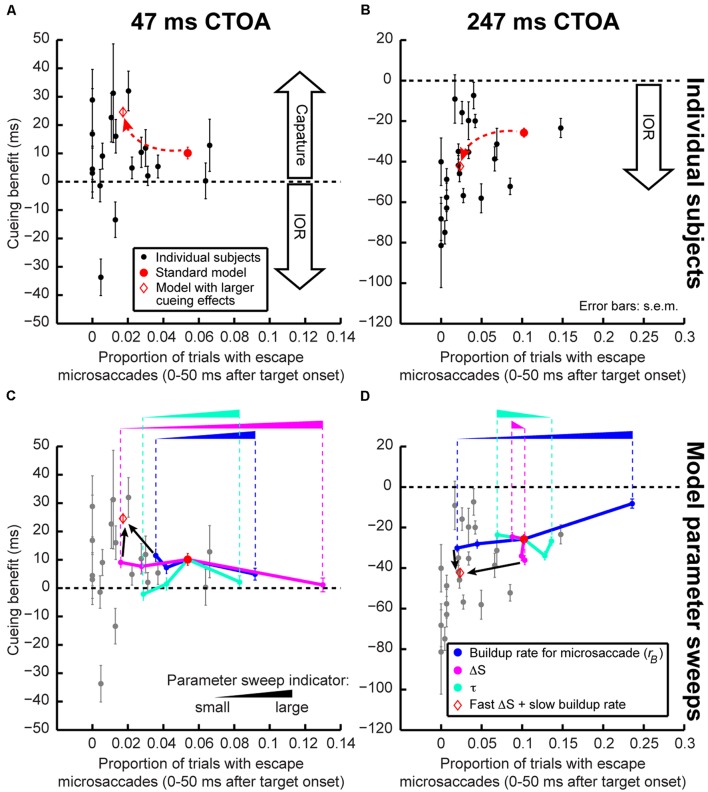
**Exploring individual subject variability. (A,B)** In each panel, we plotted each subject’s cueing benefit (as defined in **Figure 3B**) as a function of his/her proportion of trials with “escape” microsaccades <50 ms after target onset. Each dot represents a subject, and each panel shows his/her performance at one CTOA. The red circle shows model performance when run as in Section “Results,” and the red diamond shows it when run with fast ΔS (*μ_stimulus_* 1/4 of the standard model) and slow buildup rate (*k_m_* 1/4 of the standard model, see Materials and Methods). Cueing effects were stronger for lower “escape” microsaccade frequency. With simple parameter changes, the model could exhibit similar changes. **(C,D)** Model parameter sweeps allowing us to explore model robustness. Starting from the standard model (red circle), we changed one parameter at a time while holding all other parameters constant. We changed the dominant buildup rate (*k_m_*, blue), the dominant ΔS processing delay (*μ_stimulus_*, magenta), or the countermanding time constant (τ, cyan). Model performance moved in systematic ways as parameters changed (the latter were indicated by the ramp icon where the height of a ramp correlates with the size of the parameter being swept). For short CTOA’s **(C)**, ΔS was a primary determinant of performance changes. For long CTOA’s **(D)**, buildup rate played a prominent role. When two parameters were changed at a time (red diamond with the smallest *μ_stimulus_* and *k_m_*), the model moved along a non-linear trajectory like our subjects. Error bars denote SEM. The gray dots in **(C,D)** show individual subject data for easier comparison to the model trajectories. In each parameter set, the model was run for 2,000 trials.

To further investigate the above idea, we asked whether our model could help us understand inter-individual differences in cueing dynamics. We reasoned that subjects with different microsaccadic rhythms might exhibit different cueing effects. For each subject and CTOA, we measured the frequency of trials containing “escape” microsaccades within 50 ms after target onset. Since “escape” microsaccades depend on intrinsic microsaccadic rhythm dynamics (i.e., how fast the rise-to-threshold process operates), this allowed us to relate each subject’s individual microsaccade dynamics to his/her cueing effects. Across subjects, there was a (seemingly non-linear) relationship between “escape” microsaccade frequency and cueing-effect magnitude: the fewer the “escape” microsaccades, the stronger the cueing effects (**Figures 8A,B**). Our model captured this relationship when buildup rate and sensory-processing delay, ΔS, were altered. This second model exhibited both fewer “escapes” and stronger capture/IOR (**Figures 8A,B**). Thus, simple parameter changes captured the apparently complex relationships between microsaccadic rhythms and cueing effects across individuals.

The above parameter-change exercise proved particularly useful when we extended it even further, in order to understand which model parameters were most important for altering individual subject performance. Starting from the standard model (red circle in **Figure 8**), we held all parameters constant and changed only microsaccade buildup rate, sensory-processing delay after cue/target onset (ΔS), or the time constant of countermanding/phase-resetting (τ). The results of these parameter sweeps are shown in **Figures 8C,D**. In each sweep, the parameter being swept was moved to 1/4, 1/2, or 2 times the parameter in the standard model. At the shortest CTOA (47 ms), there was a large reduction in “escape” microsaccades and a concomitant increase in attentional capture when ΔS was reduced (**Figure 8C**, magenta). Sweeps of microsaccade buildup rate (blue) or countermanding dynamics (τ, cyan) gave comparatively smaller changes, especially for “escape” frequency (**Figure 8C**). This makes sense intuitively because for attentional capture, the time between cue and target onset is so short that ΔS, which jumpstarts the whole resetting process, would be expected to have maximal impact. This result is also consistent with a possible role of short-latency SC visual bursts in phase resetting (Hafed and Ignashchenkova, 2013). In contrast, at maximal IOR (247 ms), microsaccade accumulator buildup rate (**Figure 8D**, blue) had the biggest impact: slower buildup rates decreased “escape” microsaccade frequency and increased IOR. Again, in retrospect, this is intuitive because buildup rate affects how often microsaccades occur (i.e., microsaccade temporal frequency); with slower rhythms, it is easier (on average) to reset microsaccades by target onset (e.g., **Figure 5C**) because the accumulator would generally be lower than the range at which “escape” microsaccades occur (Hafed and Ignashchenkova, 2013). ΔS and τ had a weaker impact on model “escapes” at 247 ms CTOA. Interestingly, changing both buildup rate and ΔS together was sufficient to move the model in a non-linear direction consistent with the direction exhibited by individual subjects (**Figures 8C,D**; red diamond).

It is interesting to note that by altering the strength of attentional capture and IOR in **Figure 8**, our model parameter sweeps were in reality also changing the time point at which performance flipped from facilitation to cost after cue onset. That is, our model was able to replicate conditions in which the dynamics of **Figures 3B,E** might appear slower than in our current experiments. Moreover, our model parameter sweeps also covered large ranges. This not only suggests that our model is robust, but it also suggests that the model can be useful for predicting performance changes under large brain perturbations (e.g., with pharmacology). Finally, our model parameter sweeps allowed us to estimate ranges of microsaccade temporal frequency that would be expected in experiments. We converted the baseline (i.e., pre-cue) microsaccade frequency measurements of **Figure 8** into temporal rates, and we found that our standard model exhibited 3 Hz microsaccadic rhythms, and our slower one exhibited 1.5 Hz.

### Microsaccades Influence Behavior Irrespective of Prior Cueing and in Different Response Modalities

A strong prediction of our model (**Figure 2C**; see Materials and Methods) is that instantaneous microsaccadic planning state at target onset should be sufficient to modulate orienting efficacy (i.e., the response accumulator), and independently of prior cueing: if a peripheral target appears when microsaccades are prepared in one direction, orienting efficacy (response accumulator slope) would be higher than if movements are prepared opposite (**Figures 1C and 2C**), and this is a function of peri-microsaccadic changes in visual sensitivity (Hafed, 2013; Chen et al., 2015).

Psychophysically, the only measurable indicator of instantaneous microsaccadic planning direction at target onset is the direction of “escape” microsaccades (Hafed and Ignashchenkova, 2013). For these microsaccades, the accumulator (**Figure 2**) is high enough to escape cancelation by the target, and the occurring microsaccades thus reveal the underlying microsaccadic spatial direction that existed at target onset. Based on our model, we predicted that even without cueing, final saccade RT on trials with “escape” microsaccades toward the target would be faster than RT with away “escapes” (because the target appears in the pre-microsaccadic interval in which differential direction effects occur; **Figure 1**). We used a simple saccade RT task with *no* prior cueing to test this (see Materials and Methods). We found that RT was indeed faster if the “escape” microsaccade was toward the target than if it was opposite, consistent with our model prediction and recent neurophysiology (Chen et al., 2015). In monkey P, RT on trials with a target-directed “escape” was 211 ms but 222 ms when the “escape” was opposite (*p* = 0.0148, two-sided *t*-test, *N* = 232 trials for same, *N* = 235 for opposite). The same effect was observed in monkey N (154 ms < 164 ms for same and opposite “escape” directions, *p* = 0.0064, two-sided *t*-test, *N* = 271 for same, *N* = 237 for opposite), as well as an additional human subject. Thus, independently of prior cueing, current microsaccadic rhythms at target onset had measurable impacts on RT to a peripheral saccade target.

**FIGURE 9:**
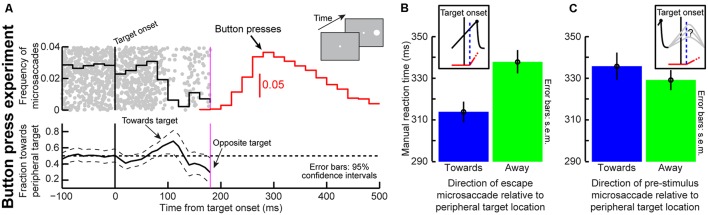
**An influence of microsaccades on manual RT’s. (A)** Microsaccade frequency (top panel) and direction (bottom panel) in a simple fixation task (inset). Human subjects fixated and pressed a button as fast as possible when a target appeared. Near target onset, microsaccade frequency behaved similarly to microsaccade frequency near target onset in our earlier cueing task with saccades as the response modality (**Figure 4**). Microsaccade direction also behaved similarly, showing an early bias toward the target and then a later bias opposite (Hafed and Ignashchenkova, 2013). *N* = 3,628 trials. All conventions are similar to **Figure 4A**. **(B)** We tested the prediction that current microsaccadic planning phase at target onset influences manual RT’s (see **Figure 1**). We analyzed manual RT on trials with target onset before microsaccades in different directions (see Materials and Methods). Manual RT was faster if the target was congruent with upcoming microsaccade direction (blue) than if it was opposite (green; *p* = 0.0015, *t*-test, *N* = 187 same, *N* = 192 opposite). **(C)** This effect disappeared when the microsaccade ended before target onset (*p* = 0.412, *N* = 214, *N* = 210). This is so because there is no differential effect of microsaccade directions in the post-movement interval (Chen et al., 2015; **Figure 1B**). Error bars denote 95% confidence intervals in **(A)** and SEM. in **(B,C)**.

Similar observations should also occur when manual responses are used to detect targets, because it is target-related visual bursts that influence final RT independent of response modality (Sapir et al., 1999; Fecteau et al., 2004; Hafed et al., 2015). We thus performed a second simple RT experiment (i.e., *without* cueing) but with button presses (**Figure 9A**). We asked eight human subjects to press a button as soon as a target appeared, and without any prior cueing (see Materials and Methods). We measured microsaccade frequency and direction around target onset and found virtually indistinguishable patterns from those in our original cueing task with saccades (compare **Figure 9A** to **Figure 4A** around target onset). Most importantly, on trials with “escape” microsaccades toward the target, manual RT’s were significantly faster than when the “escapes” were opposite the target (**Figure 9B**, *p* = 0.0015, two-sided *t*-test, *N* = 187 trials for same, *N* = 192 for opposite). If microsaccades had ended before target onset, meaning that the instantaneous microsaccadic spatial phase at target onset was uncertain (**Figure 9C**, inset), the effect disappeared (*p* = 0.412, two-sided *t*-test, *N* = 214 for same, *N* = 210 for opposite). This is also consistent with the lack of directional effects in the post-microsaccadic interval (**Figure 1B**).

Thus, regardless of prior cueing, the presence of microsaccades near target onset has measureable impacts on RT, whether with saccades or manual presses. Combined with known changes in microsaccade times and directions after cue onset using manual responses (Hafed and Clark, 2002; Engbert and Kliegl, 2003; Hafed et al., 2011; Hafed, 2013) and recent neurophysiology (Chen et al., 2015), all of these results suggest that our framework can account for both classic ways of studying IOR (with saccade or manual RT’s), and that a primary determinant of attentional capture or IOR effects may be the instantaneous state of ongoing microsaccadic activity at which targets appear.

### Post-cue Microsaccades Reflect Oculomotor Control Over Foveal Motor Error

**FIGURE 10:**
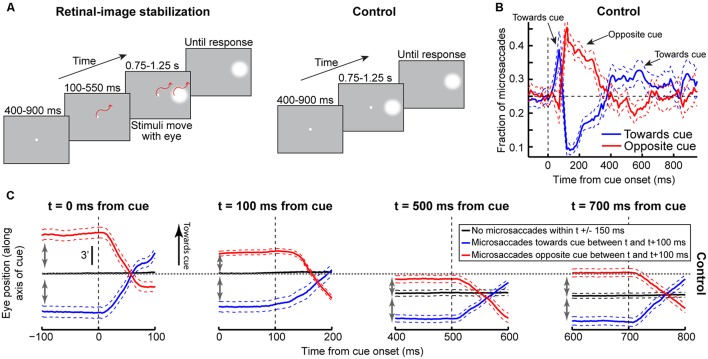
**Understanding why post-cue microsaccades directionally oscillate. (A)** Cue onset with retinal-image stabilization was compared to a control condition (see Materials and Methods). **(B)** The proportion of microsaccades in the control condition whose directions were <45° from the direction of the cue (blue) or opposite it (red). Microsaccades oscillated as in **Figure 4**. Error bars denote 95% confidence intervals. **(C)** We hypothesized that these oscillations primarily reflected eye-position error corrections. We thus analyzed such error at different times. For each time *t*, we measured average eye position when there were no microsaccades within *t*±150 ms. This was deemed the preferred retinal locus during stable fixation at *t* (black). We then measured average eye position when there were microsaccades between *t* and *t*+100 ms toward (blue) or opposite (red) the cue. Regardless of *t*, microsaccades toward the cue occurred when previous eye position error from the preferred retinal locus was away from the cue (compare blue to black in the interval before *t*); microsaccades opposite the cue occurred when previous eye position error was toward the cue (compare red to black). This held when most microsaccades according to **(B)** were either toward the cue (*t* = 0 ms), opposite the cue (*t* = 100 ms), or not particularly directional (later *t*’s). **Figure 11** provides a reason for why microsaccades oscillated despite being predominantly corrective. Error bars denote SEM.

Finally, it may be argued that, even though microsaccadic oscillations can account for cueing dynamics as per our model, these oscillations themselves might reflect peripheral covert attentional oscillations (i.e., oscillations in intrinsic visual sensitivity independently of any type of eye movement). We hypothesized instead that microsaccadic behavior reflects corrective movements of foveal motor error (Guerrasio et al., 2010; Ko et al., 2010). To test this, we ran a task (**Figure 10A**) on two monkeys implanted with scleral search coils (Judge et al., 1980). We applied retinal-image stabilization of both the fixation spot and peripheral cue, permitting us to release the reins on the eye while controlling foveal errors (see Materials and Methods), and we compared this condition to a randomly interleaved control condition. Applying this sensitive eye-movement technique to our human subjects would not have been possible with our video-based eye tracker.

In the control condition, microsaccade directions oscillated (**Figure 10B**), consistent with our earlier results (**Figure 4**). To demonstrate that these oscillations primarily reflected corrective movements after the cue has “rocked the boat” of fixation, and independent of peripheral covert attentional oscillations, we analyzed eye position error at different times relative to cue onset. For each time window that we analyzed (**Figure 10C**), we compared eye position in trials in which no microsaccades occurred to trials in which microsaccades occurred either toward or opposite the cue. Regardless of the time window, microsaccades toward the cue were predominantly triggered to reduce a foveal motor error away from the preferred retinal locus and opposite the cue (**Figure 10C**, blue). Similarly, microsaccades opposite the cue were predominantly triggered when the eye was deviated in cue direction (**Figure 10C**, red). Thus, even with cue onset, the primary determinant of microsaccade directions was foveal motor error from the preferred retinal locus.

**FIGURE 11:**
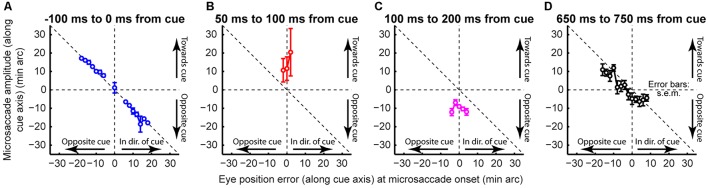
**Rocking the boat of fixation.** Microsaccade direction as a function of instantaneous eye position error at the time of microsaccade onset. **(A)** During steady fixation, microsaccades were corrective. **(B)** Immediately after cue onset, microsaccades were larger than position error (Hafed and Ignashchenkova, 2013). This contributed to getting subsequent movements opposite the cue to correct for the overshoot **(C)**. By a long time after cue onset **(D)**, eye movements were corrective once again. The oculomotor system acted like an underdamped control system in the face of cue onset. Error bars denote SEM.

If that is the case, then why does cue onset cause such coherent direction oscillations at all? According to our model, the cue was most likely to elicit an early “escape” microsaccade in its direction if a microsaccade was already being planned in that direction (i.e., if the microsaccade accumulator in **Figures 2B,C** was already high enough near threshold). Such priming would be most likely if eye position error favored generating a corrective saccade in the same direction, explaining why early microsaccades after cues in **Figure 10C** (*t* = 0 ms) were still primarily corrective. However, these eye movements must overshoot in order to give rise to coherent post-cue direction oscillations, as was also suggested recently (Hafed and Ignashchenkova, 2013). This is indeed what we found (**Figure 11**). Before cue onset, most microsaccades were corrective for eye position error (**Figure 11A**). However, within a narrow time window in which post-cue microsaccades were predominantly in the cue direction (e.g., **Figure 10B**), triggered microsaccades were larger than position error (**Figure 11B**). This contributed to getting subsequent movements opposite the cue (**Figure 11C**). Thus, the cue “rocked the boat of fixation,” requiring oculomotor rebalancing (Goffart et al., 2012), and the oculomotor system acted like an underdamped control system that was oscillating as it controlled eye position. By a long time after cue onset (**Figure 11D**), eye movements were corrective once again. Note that in **Figure 11D**, the preferred retinal locus became located slightly away from the cue location (i.e., the center of mass of the data was shifted to negative x-axis locations). This observation (routinely seen in covert attention studies that analyze eye movements), suggests that the oculomotor system favors the foveal stimulus more than the peripheral one, to the extent that it “leans” the other way to rebalance fixation.

**FIGURE 12:**
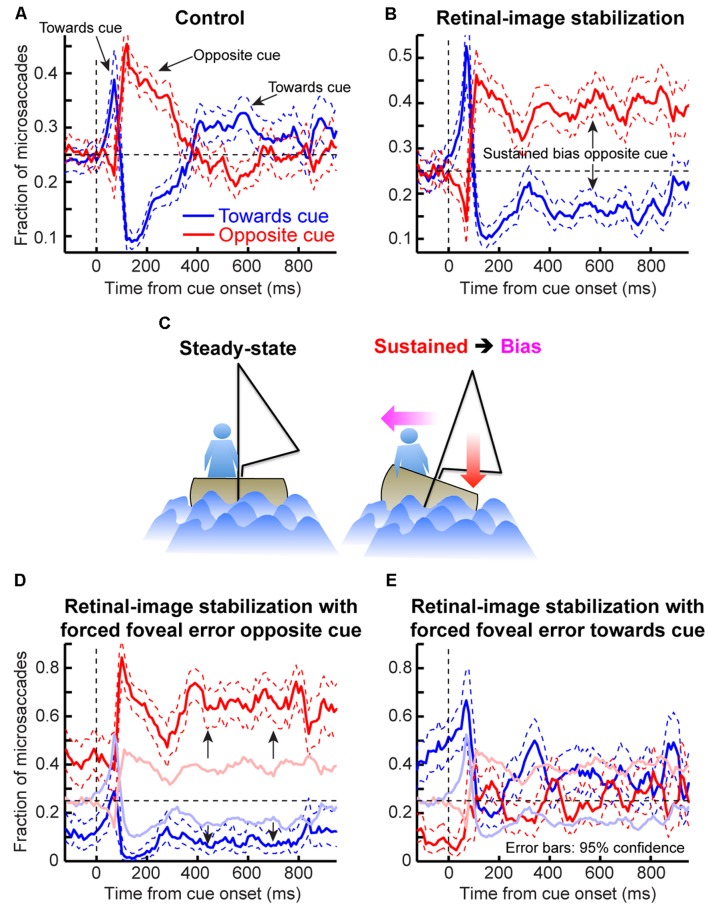
**Disrupting post-cue microsaccade direction oscillations by simply controlling foveal motor error. (A)** Microsaccade directions in the control condition (repeated from **Figure 10B** to facilitate comparison to the other panels). **(B)** With retinal-image stabilization, after the initial cue-directed “escapes,” microsaccades became constantly biased opposite the cue. This is analogous to a disturbed “fixation balance” (Goffart et al., 2012). A persistent peripheral cue causes an imbalance in the oculomotor system that is rebalanced if persistent saccades are generated in the opposite direction. **(C)** Analogy explaining **(B)** and the idea of fixation balance (Goffart et al., 2012). A sustained, not transient, wind results in a sustained sailor bias. Note that this is an exacerbation of the preferred retinal locus bias away from the cue that we saw after cue onset in the control condition of **Figure 10C** (*t* = 500 and 700 ms from cue) and **Figure 11D** (see Results). **(D,E)** Similar to **(B)** but when forcing the fixation spot ∼2.7′ away from gaze [either opposite the cue **(D)** or toward it **(E)**]. Microsaccade direction was strongly influenced by foveal motor error, and in all cases, the control oscillations were disrupted. Thus, oscillations in microsaccade directions after cue onset reflect optimization of eye position during fixation. The faint colors are those in **(B)** but included to facilitate comparison. Error bars denote 95% confidence intervals.

The above results are consistent with the idea that cue onset merely *unmasks* the properties of an ongoing oculomotor mechanism to control and optimize eye position on the fixation spot. We also analyzed microsaccade directions during retinal-image stabilization, and further established that post-cue microsaccadic oscillations were primarily dictated by foveal error. During stabilization trials, in which we experimentally controlled instantaneous foveal error, microsaccade direction oscillations were strongly disrupted: after the initial cue-directed “escapes” predicted by our model and (Hafed and Ignashchenkova, 2013), microsaccades had a sustained bias opposite the cue (**Figure 12B**; compare to **Figure 12A**). This is an expected consequence of a disturbed fixation equilibrium (Goffart et al., 2012), which can again be explained by the analogy that cues “rock the boat” of fixation (**Figure 12C**; a sustained, rather than transient, wind would result in a sustained sailor bias after her initial perturbation). In fact, even in the control condition, the preferred fixation position after cue onset became slightly biased away from the peripheral cue (compare black curves of **Figure 10C** at different times after cue onset; also **Figure 11D**): in the face of a peripheral stimulus, the oculomotor system avoided orienting toward that stimulus by leaning the other way (**Figure 12C**), and retinal-image stabilization exacerbated this effect. Finally, we also applied retinal-image stabilization but now with a forced foveal error (∼2.7′) either opposite (**Figure 12D**) or toward (**Figure 12E**) the cue. In these cases, and consistent with the above analyses, microsaccade directions were very strongly modulated by foveal motor error.

Thus, all of the above analyses suggest that direction oscillations under normal fixation might reflect corrective eye movements that are cue-reset, and not necessarily peripheral covert attentional oscillations.

## Discussion

We embarked on a theoretical investigation of the impacts of peri-microsaccadic changes in vision on Posner cueing. We found that a simple model invoking motor repetitiveness and pre-microsaccadic alteration of vision (**Figure 1**) *sufficiently* accounts for attentional capture and IOR. Unlike other models, at the time of dictating its final behavior, our model knows nothing about the previous cue location or what top–down covert attentional strategies are needed. All it does is react to stimuli (like wind gusts, **Figure 2D**), with the spatial and temporal phase of these stimuli determining how efficient the response to them is. Moreover, microsaccade direction oscillations, a critical component of the model, reflect oculomotor control over foveal error (Guerrasio et al., 2010; Ko et al., 2010), independent of peripheral covert attentional oscillations.

The physiological implications of our work are intriguing, especially in light of prior IOR research. In the SC, target-related visual bursts are enhanced for short CTOA’s and suppressed for longer ones (Dorris et al., 2002; Fecteau et al., 2004; Fecteau and Munoz, 2005). According to our model, such modulations should be *synchronized* with microsaccades, and independent of cueing. This is indeed what we found in both SC and FEF (Chen et al., 2015): without cueing, stimulus onsets before microsaccades elicit enhanced visual bursts if microsaccade directions are congruent with stimulus location and suppressed bursts if microsaccades are incongruent. These results suggest that peri-microsaccadic changes in vision (Zuber and Stark, 1966; Herrington et al., 2009; Hafed and Krauzlis, 2010; Hafed, 2013; Chen et al., 2015), coupled with microsaccade repetitiveness, may be *sufficient* to account for Posner cueing effects.

Our model is particularly attractive because it provides a parsimonious, yet mechanistic, way for understanding Posner cueing. Indeed, our framework does not deny the “need” for attention in general, since it is already well-known that a tight link exists between attention and saccade motor preparation (Hafed et al., 2015). According to the classic pre-motor theory of attention (Sheliga et al., 1994; Kustov and Robinson, 1996) and the re-entry hypothesis (Hamker, 2003, 2005), as well as some earlier IOR accounts (Rafal et al., 1989), saccade preparatory signals are expected to modulate visual representations and performance. Since microsaccades are generated using similar mechanisms to larger saccades (Hafed et al., 2009; Hafed, 2011), and since microsaccades occur repetitively (Gaarder et al., 1966; Bosman et al., 2009; Hafed and Ignashchenkova, 2013), it stands to reason that fixation might be periodically interspersed by changes in performance as a function of microsaccade generation (Hafed, 2013; Chen et al., 2015; Hafed et al., 2015). Thus, our model merely integrated two basic concepts (peri-saccadic modulations and repetitive saccade generation; **Figure 1**) to provide a simple, yet mechanistic, account of cueing effects. We believe that this account is especially relevant given emergent evidence of a fundamental role of the SC in influencing behavior (Lovejoy and Krauzlis, 2010; Zenon and Krauzlis, 2012; Krauzlis et al., 2013, 2014). In this evidence, manipulating activity in the SC, a structure critical for saccade generation, dramatically altered attentional performance even when cortical neuronal activity was unaffected. Since microsaccades are associated with altered SC activity (Hafed et al., 2009, 2013; Hafed and Krauzlis, 2012), microsaccade generation might be expected to contribute to behavioral cueing effects.

These recent SC results, coupled with earlier evidence (Sapir et al., 1999; Ignashchenkova et al., 2004), also suggest that our results can generalize to performance measures beyond RT. Indeed, attentional oscillations using visual acuity as the perceptual measure are also synchronized to microsaccades (Hafed, 2013). These results also suggest that our work can be reconciled with observations of behavioral and neural oscillations during attentional allocation (e.g., Busch and VanRullen, 2010; Drewes and VanRullen, 2011; Landau and Fries, 2012; Fiebelkorn et al., 2013; VanRullen, 2013; Vinck et al., 2013; Song et al., 2014). This is so because the saccadic system synchronizes with various brain rhythms (e.g., alpha; Gaarder et al., 1966) and also undergoes “entrainment” with rhythmic stimuli (West and Boyce, 1968). In fact, IOR was recently viewed as a manifestation of subsampled behavioral oscillations, which reflect multiple brain rhythms (Song et al., 2014). Our framework, in combination with these previous works, as well as our own neuronal observations (Chen et al., 2015), suggests a novel way for interpreting such perceptual alterations normally attributed to covert attention.

Generalization of our framework is also possible for other motor outputs and stimulus configurations. For example, IOR is often studied with manual RT’s, which does not conflict with our results (**Figure 9**). This is not surprising because at the level of the SC, attentional capture and IOR are visual burst phenomena (Dorris et al., 2002; Fecteau et al., 2004; Fecteau and Munoz, 2005; Boehnke and Munoz, 2008), which then translate into either manual (Sapir et al., 1999) or saccadic (Dorris et al., 2002) effects. In terms of stimulus configurations, IOR is observed at the midpoint between two simultaneous cues (Christie et al., 2013). This is consistent with our recent observation that “escape” and subsequent microsaccades oscillate along the “vector average” axis when simultaneous cues are presented (Hafed and Ignashchenkova, 2013). Moreover, when tasks become perceptually difficult, microsaccade rhythms are altered (Pastukhov and Braun, 2010; Hafed et al., 2011), which could account for different temporal patterns of IOR (e.g., Castel et al., 2005). Related to such alteration, during endogenous attentional conditions, in which behaviorally relevant locations are identified and maintained for prolonged periods of time, microsaccades also show direction biases to these locations, and they are associated with microsaccade-time-locked performance changes similar to those predicted by our model (Hafed et al., 2011, 2013). Thus, the links between microsaccades and attention suggested by our model might also extend to more endogenous cueing conditions than the ones we explored here. Finally, when spatial working memory becomes involved, small “memory” scan paths of microsaccades could be envisioned that can explain apparent working memory effects (Dodd et al., 2003).

**FIGURE 13:**
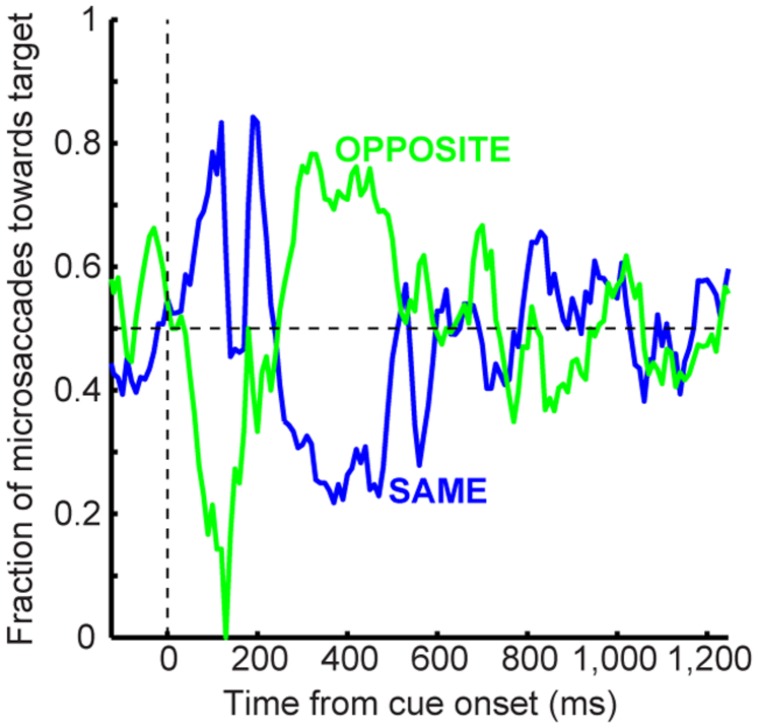
**Persistence of anti-phase modulations in the saccadic system after cue onset between the “same” and “opposite” conditions.** This data shows the same data as in **Figure 4A** (bottom) from our human experiments, but drawn in an alternative fashion (similar to **Figure 4C**). The figure plots microsaccade directions from the 1,247 ms CTOA trials. Each curve shows the fraction of microsaccades directed toward the target location. Blue is from trials with the target in the same cued location, and green from opposite trials. The resetting operation of the cue caused direction oscillations (first toward the cue and then opposite). Because the cue was in different locations across the “same” and “opposite” conditions, these cue-induced oscillations were anti-phase with respect to each other, and the anti-phase relationship persisted for a long time, albeit with a weaker magnitude (note time points beyond 600 ms). This is an expected outcome of our model: the anti-phase relationship between successive microsaccades persists continuously, but evidence for it is eventually lost because of jitter in inter-saccadic intervals/directions. Importantly, the observation of apparent persistent anti-phase correlations even 1 s after cue onset might also explain why IOR can be long lasting.

A significant premise of our model is that saccades contain temporal structure (Gaarder et al., 1966; Bosman et al., 2009; Hafed and Ignashchenkova, 2013). It may be asked, given this, why plots of microsaccade frequency do not show volleys of frequency peaks, as might be expected from a purely harmonic oscillation? This question also applies to microsaccade directions, which eventually lose lock to the cue and look random (e.g., **Figures 4A,D**). This happens because of jitter in inter-saccadic intervals and directions. In our data, we had even more evidence for persistent oscillations. For example, **Figure 13** plots microsaccade directions from our human subjects in the cueing task but drawn in a manner similar to **Figure 4C**. This way of plotting the data highlights the anti-phase relationship between “same” and “opposite” conditions. Importantly, the figure reveals that microsaccade directions in the two conditions remained relatively anti-phase with respect to each other even up to ∼1,000–1,200 ms, which could contribute to observations that IOR can persist for long CTOA’s (**Figure 3**). Thus, there was a long lasting effect of cue-induced phase resetting. Moreover, **Figure 13** shows that the anti-phase relationships appeared to persist across different frequency bands. Thus, future analysis of the spectral content of these time courses might reveal evidence of cross-frequency coupling as was recently done for RT itself (Song et al., 2014).

Concerning the important role of foveal error that we uncovered, we think that it suggests that foveal processing in general, even with a mere fixation spot, can have substantial contributions to behavioral and neuronal performance changes observed in tasks that are not originally designed to investigate foveal vision. In retrospect, this is not too surprising because there is large magnification of foveal vision in many brain areas. In addition, as far as the oculomotor and visual systems are concerned, the primary task in many paradigms with a fixation spot (like Posner cueing) is to successfully maintain gaze at that spot, independent of peripheral events, and independent of the experimenter’s goal.

Finally, our results highlight two intriguing, but often neglected, observations. First, most IOR studies do not track eye movements (either at all or accurately enough), and they use large fixation regions (e.g., 1-deg squares). Given that we found an influence of even 2.7′ foveal error on microsaccade patterns, and given that microsaccades can have a substantial influence on visual analysis (Hafed and Krauzlis, 2010; Hafed, 2013; Chen et al., 2015; Hafed et al., 2015), our results bring to caution a massive need to consider the role of tiny eye movements in attention studies. More seriously, while the Posner paradigm is appealing due to its simplicity, it uses stimulus events that are suited to drive a privileged “express” orienting pathway from retinal input to motor output (Wurtz and Albano, 1980; Boehnke and Munoz, 2008; Corneil and Munoz, 2014). In fact, cueing influences microsaccades with latencies significantly shorter than when most cortical visual areas implicated in perception and cognition would be activated (e.g., **Figure 4**; also Hafed and Ignashchenkova, 2013). It is thus not surprising that a model bypassing a potential role for these high-level processes can still exhibit attentional capture and IOR.

The second intriguing observation is that even though cueing rebalances performance across space (altering performance in “same” versus “opposite” locations), it also has large absolute costs. For example, in the short CTOA’s (47–247 ms), our subjects, despite showing modulations in “cueing benefit,” often exhibited very high RT’s (**Figure 3A**). These RT’s were higher than normal saccadic RT’s without cueing (Fischer and Ramsperger, 1984) and are more representative of SC inactivation (Hikosaka and Wurtz, 1985). Thus, in absolute value, the CTOA’s that presumably demonstrate “attentional capture” actually cause a significant performance cost; subjects were much better off without any cue. We think that this is a consequence of a double reset event (first by the cue and then by the immediately appearing target). This aspect of performance modulation by cueing (i.e., in absolute values of orienting efficacy rather than relative benefits/costs) is not receiving much scrutiny, but it is one that we believe is worthy of note.

## Author Contributions

XT, MY, and ZH implemented the human experiments and model. MY and ZH performed the retinal-image stabilization experiments. ZH wrote the paper.

## Conflict of Interest Statement

The authors declare that the research was conducted in the absence of any commercial or financial relationships that could be construed as a potential conflict of interest.

## References

[B1] Betta E. Galfano G. Turatto M. (2007). Microsaccadic response during inhibition of return in a target-target paradigm. *Vision Res.* 47 428–436. 10.1016/j.visres.2006.09.010 17087989

[B2] Boehnke S. E. Munoz D. P. (2008). On the importance of the transient visual response in the superior colliculus. *Curr. Opin. Neurobiol.* 18 544–551. 10.1016/j.conb.2008.11.004 19059772

[B3] Bosman C. A. Womelsdorf T. Desimone R. Fries P. (2009). A microsaccadic rhythm modulates gamma-band synchronization and behavior. *J. Neurosci.* 29 9471–9480. 10.1523/JNEUROSCI.1193-09.2009 19641110 PMC6666524

[B4] Busch N. A. VanRullen R. (2010). Spontaneous EEG oscillations reveal periodic sampling of visual attention. *Proc. Natl. Acad. Sci. U.S.A.* 107 16048–16053. 10.1073/pnas.1004801107 20805482 PMC2941320

[B5] Cai R. H. Pouget A. Schlag-Rey M. Schlag J. (1997). Perceived geometrical relationships affected by eye-movement signals. *Nature* 386 601–604. 10.1038/386601a0 9121582

[B6] Carpenter R. H. Williams M. L. (1995). Neural computation of log likelihood in control of saccadic eye movements. *Nature* 377 59–62. 10.1038/377059a0 7659161

[B7] Castel A. D. Pratt J. Chasteen A. L. Scialfa C. T. (2005). Examining task difficulty and the time course of inhibition of return: detecting perceptually degraded targets. *Can. J. Exp. Psychol.* 59 90–98. 10.1037/h0087464 16035343

[B8] Chen C. Y. Hafed Z. M. (2013). Postmicrosaccadic enhancement of slow eye movements. *J. Neurosci.* 33 5375–5386. 10.1523/JNEUROSCI.3703-12.2013 23516303 PMC6704992

[B9] Chen C. Y. Ignashchenkova A. Thier P. Hafed Z. M. (2015). Neuronal response gain enhancement prior to microsaccades. *Curr. Biol.* 25 2065–2074. 10.1016/j.cub.2015.06.022 26190072

[B10] Christie J. Hilchey M. D. Klein R. M. (2013). Inhibition of return is at the midpoint of simultaneous cues. *Atten. Percept. Psychophys.* 75 1610–1618. 10.3758/s13414-013-0510-5 23877540

[B11] Corneil B. D. Munoz D. P. (2014). Overt responses during covert orienting. *Neuron* 82 1230–1243. 10.1016/j.neuron.2014.05.040 24945769

[B12] Dodd M. D. Castel A. D. Pratt J. (2003). Inhibition of return with rapid serial shifts of attention: implications for memory and visual search. *Percept. Psychophys.* 65 1126–1135. 10.3758/BF03194839 14674638

[B13] Dorris M. C. Klein R. M. Everling S. Munoz D. P. (2002). Contribution of the primate superior colliculus to inhibition of return. *J. Cogn. Neurosci.* 14 1256–1263. 10.1162/089892902760807249 12495530

[B14] Drewes J. VanRullen R. (2011). This is the rhythm of your eyes: the phase of ongoing electroencephalogram oscillations modulates saccadic reaction time. *J. Neurosci.* 31 4698–4708. 10.1523/JNEUROSCI.4795-10.2011 21430168 PMC6622921

[B15] Duhamel J. R. Colby C. L. Goldberg M. E. (1992). The updating of the representation of visual space in parietal cortex by intended eye movements. *Science* 255 90–92. 10.1126/science.1553535 1553535

[B16] Egeth H. E. Yantis S. (1997). Visual attention: control, representation, and time course. *Annu. Rev. Psychol.* 48 269–297. 10.1146/annurev.psych.48.1.269 9046562

[B17] Engbert R. Kliegl R. (2003). Microsaccades uncover the orientation of covert attention. *Vision Res.* 43 1035–1045. 10.1016/S0042-6989(03)00084-1 12676246

[B18] Fecteau J. H. Bell A. H. Munoz D. P. (2004). Neural correlates of the automatic and goal-driven biases in orienting spatial attention. *J. Neurophysiol.* 92 1728–1737. 10.1152/jn.00184.2004 15115792

[B19] Fecteau J. H. Munoz D. P. (2005). Correlates of capture of attention and inhibition of return across stages of visual processing. *J. Cogn. Neurosci.* 17 1714–1727. 10.1162/089892905774589235 16269108

[B20] Fiebelkorn I. C. Saalmann Y. B. Kastner S. (2013). Rhythmic sampling within and between objects despite sustained attention at a cued location. *Curr. Biol.* 23 2553–2558. 10.1016/j.cub.2013.10.063 24316204 PMC3870032

[B21] Fischer B. Ramsperger E. (1984). Human express saccades: extremely short reaction times of goal directed eye movements. *Exp. Brain Res.* 57 191–195. 10.1007/BF00231145 6519226

[B22] Fuchs A. F. Robinson D. A. (1966). A method for measuring horizontal and vertical eye movement chronically in the monkey. *J. Appl. Physiol.* 21 1068–1070. 4958032 10.1152/jappl.1966.21.3.1068

[B23] Gaarder K. Koresko R. Kropfl W. (1966). The phasic relation of a component of alpha rhythm to fixation saccadic eye movements. *Electroencephalogr. Clin. Neurophysiol.* 21 544–551. 10.1016/0013-4694(66)90173-8 4162884

[B24] Galfano G. Betta E. Turatto M. (2004). Inhibition of return in microsaccades. *Exp. Brain Res.* 159 400–404. 10.1007/s00221-004-2111-y 15480591

[B25] Ghitani N. Bayguinov P. O. Vokoun C. R. Mcmahon S. Jackson M. B. Basso M. A. (2014). Excitatory synaptic feedback from the motor layer to the sensory layers of the superior colliculus. *J. Neurosci.* 34 6822–6833. 10.1523/JNEUROSCI.3137-13.2014 24828636 PMC4019797

[B26] Goffart L. Hafed Z. M. Krauzlis R. J. (2012). Visual fixation as equilibrium: evidence from superior colliculus inactivation. *J. Neurosci.* 32 10627–10636. 10.1523/JNEUROSCI.0696-12.2012 22855812 PMC3473086

[B27] Guerrasio L. Quinet J. Buttner U. Goffart L. (2010). Fastigial oculomotor region and the control of foveation during fixation. *J. Neurophysiol.* 103 1988–2001. 10.1152/jn.00771.2009 20130038

[B28] Hafed Z. M. (2011). Mechanisms for generating and compensating for the smallest possible saccades. *Eur. J. Neurosci.* 33 2101–2113. 10.1111/j.1460-9568.2011.07694.x 21645104

[B29] Hafed Z. M. (2013). Alteration of visual perception prior to microsaccades. *Neuron* 77 775–786. 10.1016/j.neuron.2012.12.014 23439128

[B30] Hafed Z. M. Chen C.-Y. Tian X. (2015). Vision, perception, and attention through the lens of microsaccades: mechanisms and implications. *Front. Syst. Neurosci.* 9:167 10.3389/fnsys.2015.00167 PMC4667031 26696842

[B31] Hafed Z. M. Clark J. J. (2002). Microsaccades as an overt measure of covert attention shifts. *Vision Res.* 42 2533–2545. 10.1016/S0042-6989(02)00263-8 12445847

[B32] Hafed Z. M. Goffart L. Krauzlis R. J. (2009). A neural mechanism for microsaccade generation in the primate superior colliculus. *Science* 323 940–943. 10.1126/science.1166112 19213919 PMC2655118

[B33] Hafed Z. M. Ignashchenkova A. (2013). On the dissociation between microsaccade rate and direction after peripheral cues: microsaccadic inhibition revisited. *J. Neurosci.* 33 16220–16235. 10.1523/JNEUROSCI.2240-13.2013 24107954 PMC6618351

[B34] Hafed Z. M. Krauzlis R. J. (2010). Microsaccadic suppression of visual bursts in the primate superior colliculus. *J. Neurosci.* 30 9542–9547. 10.1523/JNEUROSCI.1137-10.2010 20631182 PMC2922969

[B35] Hafed Z. M. Krauzlis R. J. (2012). Similarity of superior colliculus involvement in microsaccade and saccade generation. *J. Neurophysiol.* 107 1904–1916. 10.1152/jn.01125.2011 22236714 PMC3331665

[B36] Hafed Z. M. Lovejoy L. P. Krauzlis R. J. (2011). Modulation of microsaccades in monkey during a covert visual attention task. *J. Neurosci.* 31 15219–15230. 10.1523/JNEUROSCI.3106-11.2011 22031868 PMC3229866

[B37] Hafed Z. M. Lovejoy L. P. Krauzlis R. J. (2013). Superior colliculus inactivation alters the relationship between covert visual attention and microsaccades. *Eur. J. Neurosci.* 37 1169–1181. 10.1111/ejn.12127 23331638 PMC4034743

[B38] Hamker F. H. (2003). The reentry hypothesis: linking eye movements to visual perception. *J. Vis.* 3 808–816. 10.1167/3.11.14 14765963

[B39] Hamker F. H. (2005). The reentry hypothesis: the putative interaction of the frontal eye field, ventrolateral prefrontal cortex, and areas V4. IT for attention and eye movement. *Cereb. Cortex* 15 431–447. 10.1093/cercor/bhh146 15749987

[B40] Herrington T. M. Masse N. Y. Hachmeh K. J. Smith J. E. Assad J. A. Cook E. P. (2009). The effect of microsaccades on the correlation between neural activity and behavior in middle temporal, ventral intraparietal, and lateral intraparietal areas. *J. Neurosci.* 29 5793–5805. 10.1523/JNEUROSCI.4412-08.2009 19420247 PMC2904875

[B41] Hikosaka O. Wurtz R. H. (1985). Modification of saccadic eye movements by GABA-related substances. I. Effect of muscimol and bicuculline in monkey superior colliculus. *J. Neurophysiol.* 53 266–291. 2983037 10.1152/jn.1985.53.1.266

[B42] Ignashchenkova A. Dicke P. W. Haarmeier T. Thier P. (2004). Neuron-specific contribution of the superior colliculus to overt and covert shifts of attention. *Nat. Neurosci.* 7 56–64. 10.1038/nn1169 14699418

[B43] Jonides J. (1981). “Voluntary versus automatic control over the mind’s eye’s movement,” in *Attention and Performance*, 9th Edn, eds Long J. B. Baddeley A. D. (Hillsdale, NJ: Erlbaum), 187–203.

[B44] Judge S. J. Richmond B. J. Chu F. C. (1980). Implantation of magnetic search coils for measurement of eye position: an improved method. *Vision Res.* 20 535–538. 10.1016/0042-6989(80)90128-5 6776685

[B45] Klein R. M. (2000). Inhibition of return. *Trends Cogn. Sci.* 4 138–147. 10.1016/S1364-6613(00)01452-2 10740278

[B46] Ko H. K. Poletti M. Rucci M. (2010). Microsaccades precisely relocate gaze in a high visual acuity task. *Nat. Neurosci.* 13 1549–1553. 10.1038/nn.2663 21037583 PMC3058801

[B47] Krauzlis R. J. Bollimunta A. Arcizet F. Wang L. (2014). Attention as an effect not a cause. *Trends Cogn. Sci.* 18 457–464. 10.1016/j.tics.2014.05.008 24953964 PMC4186707

[B48] Krauzlis R. J. Lovejoy L. P. Zenon A. (2013). Superior colliculus and visual spatial attention. *Annu. Rev. Neurosci.* 36 165–182. 10.1146/annurev-neuro-062012-170249 23682659 PMC3820016

[B49] Kustov A. A. Robinson D. L. (1996). Shared neural control of attentional shifts and eye movements. *Nature* 384 74–77. 10.1038/384074a0 8900281

[B50] Landau A. N. Fries P. (2012). Attention samples stimuli rhythmically. *Curr. Biol.* 22 1000–1004. 10.1016/j.cub.2012.03.054 22633805

[B51] Lappe M. Awater H. Krekelberg B. (2000). Postsaccadic visual references generate presaccadic compression of space. *Nature* 403 892–895. 10.1038/35002588 10706286

[B52] Lovejoy L. P. Krauzlis R. J. (2010). Inactivation of primate superior colliculus impairs covert selection of signals for perceptual judgments. *Nat. Neurosci.* 13 261–266. 10.1038/nn.2470 20023651 PMC3412590

[B53] Lupianez J. Klein R. M. Bartolomeo P. (2006). Inhibition of return: twenty years after. *Cogn. Neuropsychol.* 23 1003–1014. 10.1080/02643290600588095 21049364

[B54] Montagnini A. Chelazzi L. (2009). Dynamic interaction between “Go” and “Stop” signals in the saccadic eye movement system: new evidence against the functional independence of the underlying neural mechanisms. *Vision Res.* 49 1316–1328. 10.1016/j.visres.2008.07.018 18713642

[B55] Morris A. P. Bremmer F. Krekelberg B. (2013). Eye-position signals in the dorsal visual system are accurate and precise on short timescales. *J. Neurosci.* 33 12395–12406. 10.1523/JNEUROSCI.0576-13.2013 23884945 PMC3721846

[B56] Morris A. P. Kubischik M. Hoffmann K. P. Krekelberg B. Bremmer F. (2012). Dynamics of eye-position signals in the dorsal visual system. *Curr. Biol.* 22 173–179. 10.1016/j.cub.2011.12.032 22225775 PMC3277641

[B57] Munoz D. P. Wurtz R. H. (1995). Saccade-related activity in monkey superior colliculus. I. Characteristics of burst and buildup cells. *J. Neurophysiol.* 73 2313–2333. 7666141 10.1152/jn.1995.73.6.2313

[B58] Pastukhov A. Braun J. (2010). Rare but precious: microsaccades are highly informative about attentional allocation. *Vision Res.* 50 1173–1184. 10.1016/j.visres.2010.04.007 20382176

[B59] Pola J. (2011). An explanation of perisaccadic compression of visual space. *Vision Res.* 51 424–434. 10.1016/j.visres.2010.12.010 21192965

[B60] Poletti M. Listorti C. Rucci M. (2013). Microscopic eye movements compensate for nonhomogeneous vision within the fovea. *Curr. Biol.* 23 1691–1695. 10.1016/j.cub.2013.07.007 23954428 PMC3881259

[B61] Posner M. I. (1980). Orienting of attention. *Q. J. Exp. Psychol.* 32 3–25. 10.1080/00335558008248231 7367577

[B62] Posner M. I. Cohen Y. (1984). “Components of visual orienting,” in *Attention and Performance X*, eds Bouma H. Bowhuis D. (Hillsdale, NJ: Erlbaum), 531–556.

[B63] Posner M. I. Rafal R. D. Choate L. S. Vaughan J. (1985). Inhibition of return: neural basis and function. *Cogn. Neuropsychol.* 2 211–228. 10.1080/02643298508252866

[B64] Rafal R. D. Calabresi P. A. Brennan C. W. Sciolto T. K. (1989). Saccade preparation inhibits reorienting to recently attended locations. *J. Exp. Psychol. Hum. Percept. Perform.* 15 673–685. 10.1037/0096-1523.15.4.673 2531204

[B65] Reingold E. M. Stampe D. M. (2002). Saccadic inhibition in voluntary and reflexive saccades. *J. Cogn. Neurosci.* 14 371–388. 10.1162/089892902317361903 11970798

[B66] Rolfs M. Kliegl R. Engbert R. (2008). Toward a model of microsaccade generation: the case of microsaccadic inhibition. *J. Vis.* 8 1–23. 10.1167/8.11.5 18831599

[B67] Ross J. Morrone M. C. Burr D. C. (1997). Compression of visual space before saccades. *Nature* 386 598–601. 10.1038/386598a0 9121581

[B68] Ross J. Morrone M. C. Goldberg M. E. Burr D. C. (2001). Changes in visual perception at the time of saccades. *Trends Neurosci.* 24 113–121. 10.1016/S0166-2236(00)01685-4 11164942

[B69] Rucci M. Iovin R. Poletti M. Santini F. (2007). Miniature eye movements enhance fine spatial detail. *Nature* 447 851–854. 10.1038/nature05866 17568745

[B70] Salinas E. Stanford T. R. (2013). The countermanding task revisited: fast stimulus detection is a key determinant of psychophysical performance. *J. Neurosci.* 33 5668–5685. 10.1523/JNEUROSCI.3977-12.2013 23536081 PMC3650622

[B71] Santini F. Redner G. Iovin R. Rucci M. (2007). EyeRIS: a general-purpose system for eye-movement-contingent display control. *Behav. Res. Methods* 39 350–364. 10.3758/BF03193003 17958145

[B72] Sapir A. Soroker N. Berger A. Henik A. (1999). Inhibition of return in spatial attention: direct evidence for collicular generation. *Nat. Neurosci.* 2 1053–1054. 10.1038/15977 10570480

[B73] Satel J. Wang Z. Trappenberg T. P. Klein R. M. (2011). Modeling inhibition of return as short-term depression of early sensory input to the superior colliculus. *Vision Res.* 51 987–996. 10.1016/j.visres.2011.02.013 21354199

[B74] Sheliga B. M. Riggio L. Rizzolatti G. (1994). Orienting of attention and eye movements. *Exp. Brain Res.* 98 507–522. 10.1007/BF00233988 8056071

[B75] Sommer M. A. Wurtz R. H. (2006). Influence of the thalamus on spatial visual processing in frontal cortex. *Nature* 444 374–377. 10.1038/nature05279 17093408

[B76] Song K. Meng M. Chen L. Zhou K. Luo H. (2014). Behavioral oscillations in attention: rhythmic alpha pulses mediated through theta band. *J. Neurosci.* 34 4837–4844. 10.1523/JNEUROSCI.4856-13.2014 24695703 PMC6802725

[B77] Stampe D. M. (1993). Heuristic filtering and reliable calibration methods for video-based pupil-tracking systems. *Behav. Res. Methods Instrum Comput.* 25 137–142. 10.3758/Bf03204486

[B78] Tolias A. S. Moore T. Smirnakis S. M. Tehovnik E. J. Siapas A. G. Schiller P. H. (2001). Eye movements modulate visual receptive fields of V4 neurons. *Neuron* 29 757–767. 10.1016/S0896-6273(01)00250-1 11301034

[B79] VanRullen R. (2013). Visual attention: a rhythmic process? *Curr. Biol.* 23 R1110–R1112. 10.1016/j.cub.2013.11.006 24355791

[B80] Vinck M. Womelsdorf T. Buffalo E. A. Desimone R. Fries P. (2013). Attentional modulation of cell-class-specific gamma-band synchronization in awake monkey area v4. *Neuron* 80 1077–1089. 10.1016/j.neuron.2013.08.019 24267656 PMC3840396

[B81] West D. C. Boyce P. R. (1968). The effect of flicker on eye movement. *Vision Res.* 8 171–192. 10.1016/0042-6989(68)90005-9 5729328

[B82] Wurtz R. H. Albano J. E. (1980). Visual-motor function of the primate superior colliculus. *Annu. Rev. Neurosci.* 3 189–226. 10.1146/annurev.ne.03.030180.001201 6774653

[B83] Zenon Z. Krauzlis R. J. (2012). Attention deficits without cortical neuronal deficits. *Nature* 489 434–437. 10.1038/nature11497 22972195 PMC3448852

[B84] Zirnsak M. Steinmetz N. A. Noudoost B. Xu K. Z. Moore T. (2014). Visual space is compressed in prefrontal cortex before eye movements. *Nature* 507 504–507. 10.1038/nature13149 24670771 PMC4064801

[B85] Zuber B. L. Stark L. (1966). Saccadic suppression: elevation of visual threshold associated with saccadic eye movements. *Exp. Neurol.* 16 65–79. 10.1016/0014-4886(66)90087-2 5923485

