# Supplementary material for: A Microsaccadic Account of Attentional Capture and Inhibition of Return in Posner Cueing
Source: Front Syst Neurosci. 2016 Mar 7;10:23. doi: 10.3389/fnsys.2016.00023 (PMC4779940; doi:10.3389/fnsys.2016.00023)
Supplement: Supplementary file 1 [file Image_1.PDF]

Supplementary Material

A Microsaccadic Account of Attentional Capture and Inhibition of Return in Posner Cueing

Xiaoguang Tian, Masatoshi Yoshida, Ziad M. Hafed*

*** Correspondence:** ziad.m.hafed@cin.uni-tuebingen.de

1 Supplementary Figure

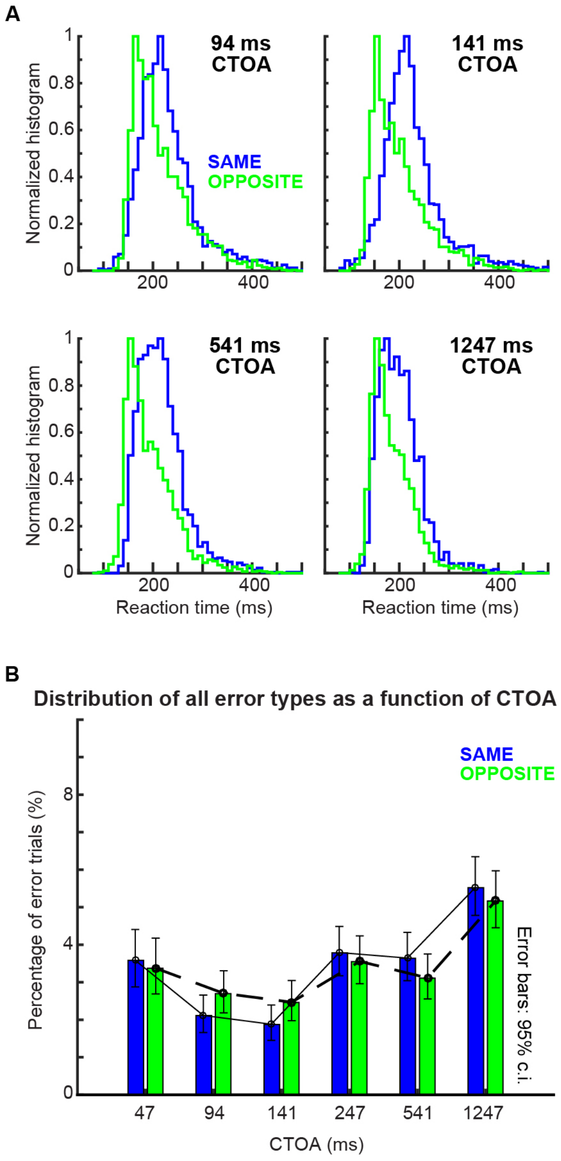

Supplementary Figure 1: (A) Reaction time (RT) distributions from each CTOA in the cueing task (Figs. 2-4). Note that RT distributions from the 47 ms and 247 ms CTOA's are shown in Fig. 4B (top) and Fig. 3C of the main text, respectively. Each panel shows RT distributions when the target appeared either in the same (blue) or opposite (green) location from the cue, and each curve was normalized by its maximum value. As can be seen, all distributions were unimodal with no anticipatory saccades (as might be expected if subjects were erroneously generating reflexive saccades to the cue instead of the target). (B) Distribution of all error types (blinks, breaks from fixation, and so on) that were excluded from analysis. The figure shows how these few error trials were distributed as a function of CTOA and cue location (same versus opposite). In each CTOA, there was no difference in the likelihood of errors between same and opposite trials. This is expected because of our 50% cue validity in the paradigm (i.e. the cue was not informative about target location). Also, errors increased modestly with longer CTOA's, which is again expected because of the prolonged fixation requirement in these conditions. Finally, note that if subjects were erroneously making reflexive saccades to the cue instead of the target in short CTOA's (e.g. 47 and 94 ms), then RT distributions for opposite trials should be heavily delayed because corrective saccades would need to be made before finally reaching the target. This is the opposite of what we found (A).
